# Congenital myasthenic syndrome due to mutations in *MUSK* suggests that the level of MuSK phosphorylation is crucial for governing synaptic structure

**DOI:** 10.1002/humu.23949

**Published:** 2019-11-25

**Authors:** Pedro M. Rodríguez Cruz, Judith Cossins, Jonathan Cheung, Susan Maxwell, Sandeep Jayawant, Ruth Herbst, Dominic Waithe, Alexandr P. Kornev, Jacqueline Palace, David Beeson

**Affiliations:** ^1^ Nuffield Department of Clinical Neurosciences University of Oxford Oxford UK; ^2^ Neurosciences Group, The John Radcliffe Hospital, Weatherall Institute of Molecular Medicine University of Oxford Oxford UK; ^3^ Department of Paediatric Neurology, Children's Hospital John Radcliffe Hospital Oxford UK; ^4^ Center for Pathophysiology, Infectiology and Immunology, Medical Science Divisions Medical University of Vienna Vienna Austria; ^5^ MRC Centre for Computational Biology and Wolfson Imaging Centre, Weatherall Institute of Molecular Medicine University of Oxford Oxford UK; ^6^ Department of Pharmacology University of California at San Diego La Jolla California

**Keywords:** AChR clustering, congenital myasthenic syndromes, dimerization, muscle‐specific kinase (MuSK), MuSK phosphorylation, neuromuscular junction, receptor tyrosine kinases, β2‐adrenergic agonists

## Abstract

*MUSK* encodes the muscle‐specific receptor tyrosine kinase (MuSK), a key component of the agrin‐LRP4‐MuSK‐DOK7 signaling pathway, which is essential for the formation and maintenance of highly specialized synapses between motor neurons and muscle fibers. We report a patient with severe early‐onset congenital myasthenic syndrome and two novel missense mutations in *MUSK* (p.C317R and p.A617V). Functional studies show that *MUSK* p.C317R, located at the frizzled‐like cysteine‐rich domain of MuSK, disrupts an integral part of MuSK architecture resulting in ablated MuSK phosphorylation and acetylcholine receptor (AChR) cluster formation. *MUSK* p.A617V, located at the kinase domain of MuSK, enhances MuSK phosphorylation resulting in anomalous AChR cluster formation. The identification and evidence for pathogenicity of *MUSK* mutations supported the initiation of treatment with β2‐adrenergic agonists with a dramatic improvement of muscle strength in the patient. This work suggests uncharacterized mechanisms in which control of the precise level of MuSK phosphorylation is crucial in governing synaptic structure.

## Graphical abstract

We report a patient with severe early‐onset congenital myasthenic syndrome and two novel missense mutations in the muscle‐specific receptor tyrosine kinase (*MUSK*; p.C317R and p.A617V). Functional studies show that p.C317R, located at the frizzled‐like cysteine‐rich domain of MuSK, disrupts an integral part of MuSK architecture resulting in ablated MuSK phosphorylation and acetylcholine receptor (AChR) cluster formation. The p.A617V substitution, located at the kinase domain of MuSK, enhances MuSK phosphorylation resulting in anomalous AChR cluster formation. This work suggests uncharacterized mechanisms in which control of the precise level of MuSK phosphorylation is crucial in governing synaptic structure.

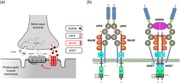

## 1 INTRODUCTION

**Figure 1:**
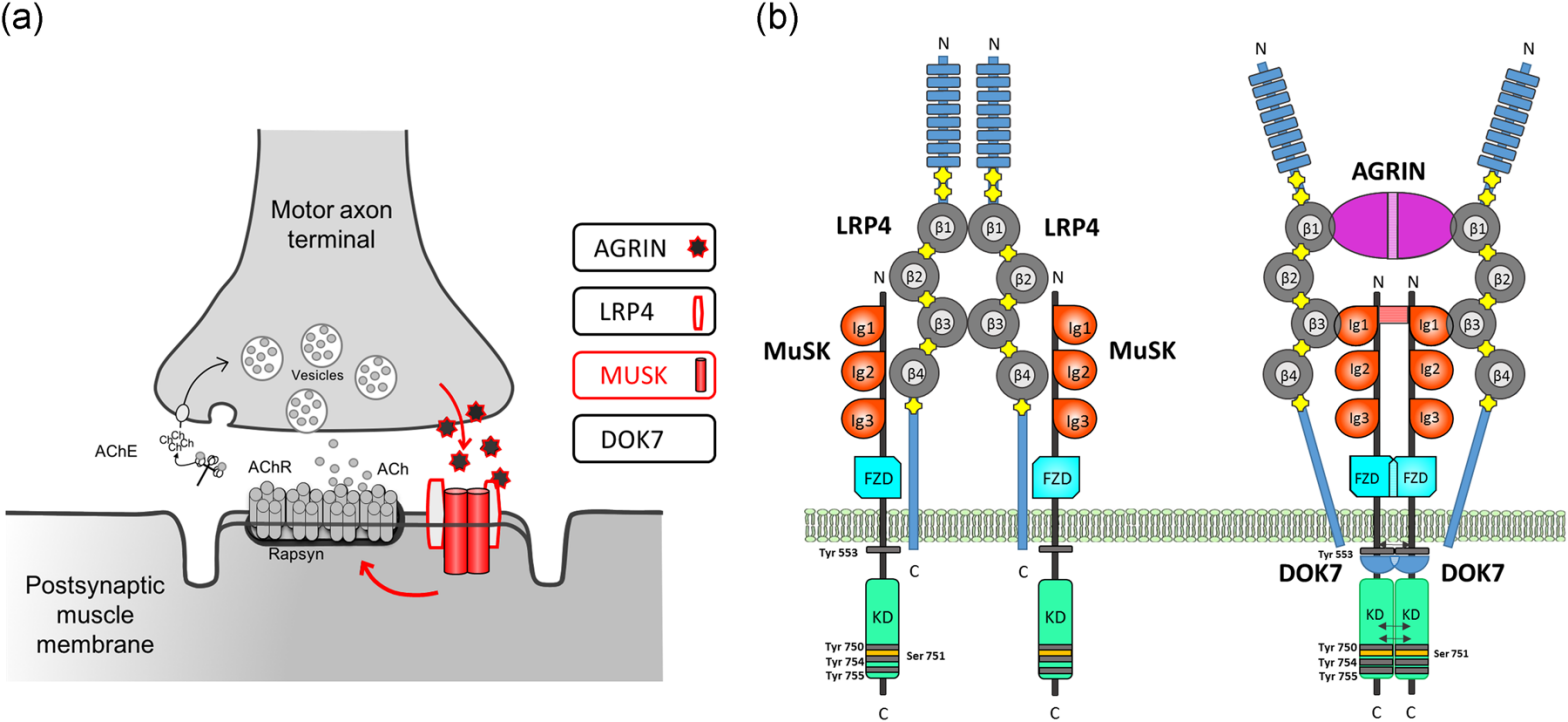
The NMJ and the agrin‐LRP4‐MuSK‐DOK7 pathway. (a) Schematic representation of the NMJ and the agrin‐LRP4‐MuSK‐DOK7 pathway. (b) The binding of agrin to the N‐terminal region of LRP4 induces a conformational change (active state) promoting the binding between LRP4 and MuSK first IgG‐like domain (Zhang, Coldefy, Hubbard, & Burden, 2011). This results in MuSK activation via dimerization and trans‐autophosphorylation of tyrosine residues within the cytoplasmic region (Schlessinger, 2000) through a not fully known mechanism. The increase in the catalytic activity creates active binding sites for other proteins such as DOK7, which amplifies the signal downstream. LRP4 is composed of eight low‐density lipoprotein Class A (LDLa) domains (blue) at the N‐terminus, followed by four YWTD β‐propeller domains (gray) bounded by epidermal growth factor‐like modules (yellow) and a short C‐terminal domain (Springer, 1998). LRP4 self‐associates and interacts with MuSK in the absence of agrin (inactive state), but is not capable of activating MuSK (Kim et al., 2008). This interaction could be important for AChRs prepatterning before innervation (Yang et al., 2001; Yumoto et al., 2012). IgG, immunoglobulin G; LRP, low‐density lipoprotein receptor‐related protein; MuSK, muscle‐specific receptor tyrosine kinase; NMJ, neuromuscular junction

The neuromuscular junction (NMJ) is a complex and highly specialized synapse between the motor neuron and the skeletal muscle fiber. Previous studies have demonstrated a critical role for the agrin‐LRP4‐MuSK‐DOK7 signaling pathway (Figure 1a) in the formation and maintenance of the NMJ (Burden, Yumoto, & Zhang, 2013; DeChiara et al., 1996; Okada et al., 2006). In particular, the phosphorylation of the muscle‐specific receptor tyrosine kinase (MuSK) plays an essential role in this pathway by orchestrating downstream events that lead to cytoskeletal reorganization (Dai et al., 2000) and clustering of acetylcholine receptors (AChRs; Wu, Xiong, & Mei, 2010). MuSK is composed of three immunoglobulin G (IgG)‐like domains, a frizzled domain (FzD), a short transmembrane domain, a juxtamembrane region (JMR), a kinase domain (KD), and a short C‐terminal tail (Hubbard & Gnanasambandan, 2013).

After its release by the nerve terminal, agrin binds to the low‐density lipoprotein receptor‐related protein 4 (LRP4) on the postsynaptic membrane forming a tetrameric complex (Zong et al., 2012). This enhances the binding of LRP4 to the first IgG‐like domain of MuSK (Zhang et al., 2012), resulting in MuSK activation via dimerization and trans‐autophosphorylation of specific tyrosine residues within the cytoplasmic region (Schlessinger, 2000; Figure 1b). Autophosphorylation of the juxtamembrane tyrosine residue in MuSK creates an active binding site for DOK7, a muscle‐specific cytoplasmic adapter of MuSK with a phosphotyrosine binding domain. The dimerization of MuSK‐associated DOK7 further stimulates MuSK kinase activity. In addition, DOK7 recruits the adapter proteins CrK and CrK‐L to the C‐terminal domain of DOK7 (Hallock et al., 2010). Finally, the signal is propagated downstream through still undefined mechanisms that eventually lead to the clustering of AChRs. MuSK can be also partially activated in the absence of agrin and this may be important for the prepatterning of AChRs before innervation (Yang et al., 2001; Yumoto, Kim, & Burden, 2012).

MuSK kinase activity is strictly regulated to limit ligand‐independent activation mainly through the JMR and the activation loop (Till et al., 2002). In addition, a serine phosphorylation site within this loop (S751) was more recently reported as a modulator of MuSK kinase activity by relieving the autoinhibition of the MuSK activation loop upon its phosphorylation (Camurdanoglu et al., 2016). The JMR of MuSK interacts with the kinase core to inhibit kinase activity. The activation loop occupies the active site cleft of the MuSK kinase domain by adopting a pseudo‐substrate conformation that impedes ATP and other substrates binding thus blocking kinase activation (Till et al., 2002). These regulatory mechanisms seem important to control postsynaptic differentiation of the NMJ (Madhavan & Peng, 2005). Upon agrin release by the nerve terminal, agrin‐mediated MuSK activation cause MuSK dimerization and trans‐autophosphorylation of Tyr553 in the juxtamembrane domain, destabilizing the juxtamembrane conformation that prevents phosphorylation of the activation loop. The subsequent transphosphorylation of the activation loop (Tyr750, Tyr754, and Tyr755) leads to full kinase activation. However, how exactly the kinase domain dimerizes and gets activated is not fully understood.

**Figure 2:**
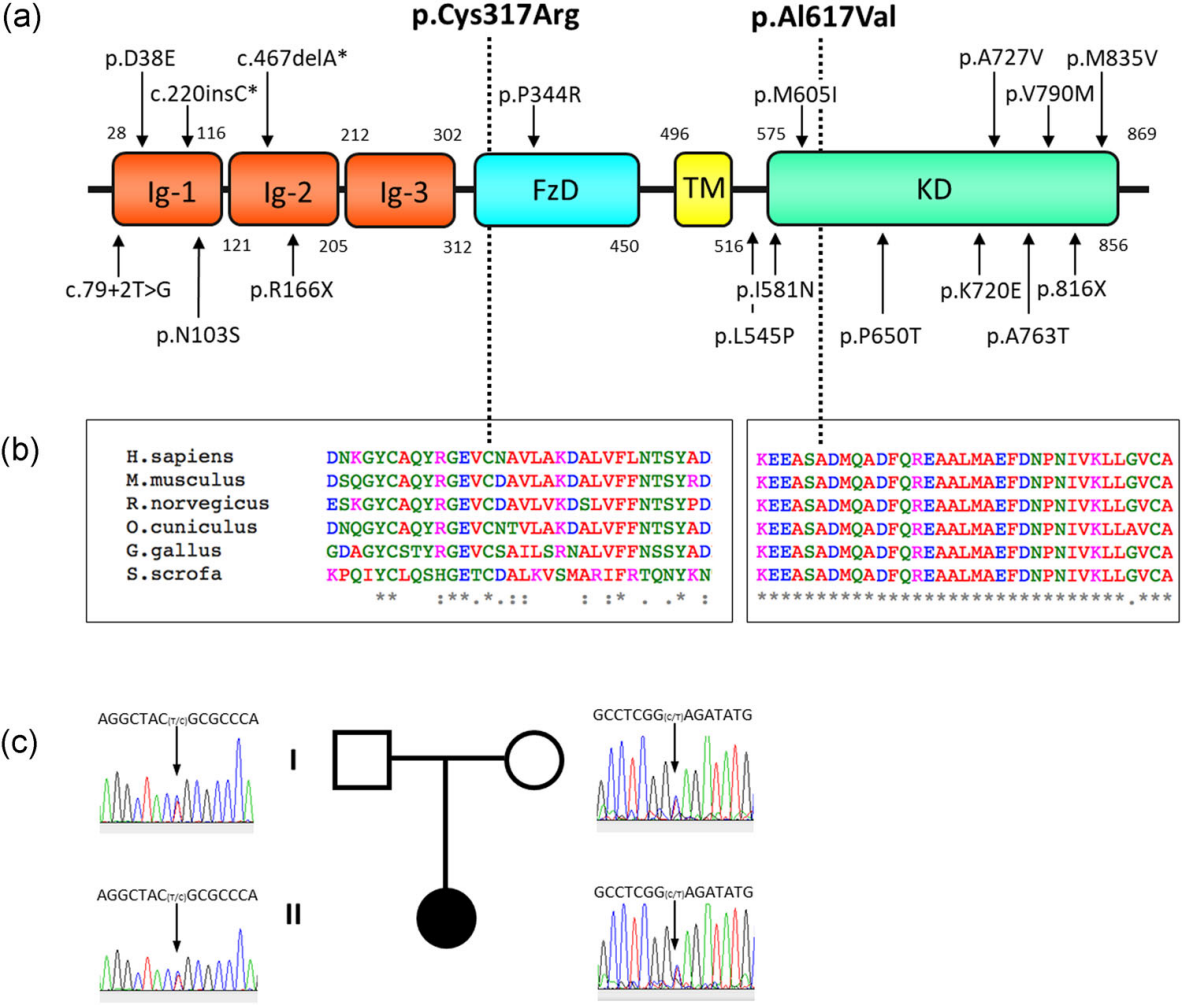
MuSK domains structure and conservation. (a) Schematic representation of MuSK drawn to linear scale and location of variants found in the patient (bold) and mutations previously reported in the literature (black). MuSK (NP_005583.1) is composed of three IgG‐like domains, a FzD, a short transmembrane domain (TMD), a JMR, a KD, and a short C‐terminal tail. (b) Protein alignments were performed using the ClustalW2 multiple sequence alignment program (http://www.ebi.ac.uk/Tools/msa/clustalw2/). (c) Segregation analysis of *MUSK* variants in the family. Pedigree symbols are shaded according to the presence of clinical symptoms. FzD, frizzled domain; IgG, immunoglobulin G; JMR, juxtamembrane region; KD, kinase domain; MuSK, muscle‐specific receptor tyrosine kinase

Mutations in *MUSK* are a known cause of congenital myasthenic syndrome (CMS), but a limited number of cases have been reported to date despite the increasing use of next‐generation sequencing (Ammar et al., 2013; Chevessier et al., 2004; Giarrana et al., 2015; Luan, Tian, & Cao, 2016; Maselli et al., 2010; Mihaylova et al., 2009; Murali, Li, Grand, Hakonarson, & Bhoj, 2019; Owen et al., 2018). More than half of *MUSK* mutations reported so far are located in the kinase domain. Others have been described in the IgG domains and less commonly in the frizzle and JMR (Figure 2a**)**. *MUSK* mutations have been shown to diminish the expression and stability of MuSK (Chevessier et al., 2004), impair MuSK‐DOK7 interaction (Maselli et al., 2010), and reduce sensitivity to agrin (Ammar et al., 2013). Here, we identify and study the underlying molecular mechanisms of impaired synaptic transmission in a patient with CMS harboring two novel missense mutations in *MUSK* (p.Cys317Arg and p.Ala617Val), so that, based on the molecular findings, an appropriate treatment strategy could be pursued.

## 2 MATERIALS AND METHODS

### 2.1 DNA sequencing and data analysis

Patient consent for DNA analysis and publication of clinical and genetic data was obtained with ethical approval from OXREC B: 04.OXB.017 and Oxfordshire REC C 09/H0606/74. Genomic DNA was isolated from the patient's and parents' peripheral blood by standard methods. Sanger sequencing was performed with primers covering exonic and flanking regions of *MUSK* (RefSeq NM_005592.3). In silico tools for the interpretation of genetic variants included Mutation Taster 2 (Schwarz, Cooper, Schuelke, & Seelow, 2014), PolyPhen‐2 (Adzhubei et al., 2010), and dbNSFP 4.0, which collates the outputs from multiple prediction algorithms and conservation scores (Liu, Jian, & Boerwinkle, 2011).

### 2.2 Generation of MuSK knock‐out C2C12 muscle cells using the CRISPR‐Cas9n system

*Musk* knock‐out (KO) C2C12 mouse muscle cells were generated with CRISPR‐Cas9 nickase genome‐editing technology via nonhomologous end joining using standard methods (Ran et al., 2013). MuSK guide oligonucleotides (Integrated DNA Technologies Inc., Coralville, IO) were designed and cloned into a modified version of plasmid pX335‐U6‐Chimeric_BB‐CBh‐hSpCas9n(D10A), a gift from Feng Zhang (plasmid; cat no. #42335; Addgene). Guide A oligonucleotide sequences were A‐Forward: 5′‐CACCGCATTCTCCCGGATGCTGTAG‐3′ and A‐Reverse: 5′‐AAACCTACAGCATCCGGGAGAATGC‐3′. Guide B oligonucleotide sequences were B‐Forward: 5′‐CACCGCTCCTCACCATTCTGAGCG‐3′ and B‐reverse: 5′‐AAACCGCTCAGAATGGTGAGGAGC‐3′. C2C12 cells were electroporated with 10 μg of each plasmid using a Neon® Transfection System (Life Technologies), selected with Geneticin® Selective Antibiotic (cat no. #10131027; Life Technologies), and cloned in 96‐well flat‐bottomed culture plates using fluorescence‐activated cell sorting. *Musk* KO screening was performed with the AChR clustering assay (Figure S1). Confirmation of *Musk* KO was conducted by Sanger sequencing after polymerase chain reaction (PCR) of genomic DNA (primer pair: 5′‐TGGTGCTTTGGTTATGGAGCC‐3′ and 5′‐GAGGAGGGGTCTAAGGCTTG‐3′) and western blot analysis of MuSK protein expression (Figure S2–4).

### 2.3 Retroviral production and infection of MuSK KO cells

The coding sequence of *MUSK* WT (ENST00000416899.7), A617V or C317R was subcloned into the retroviral vector pBabe‐PURO‐EGFP. Phoenix™‐ECO retrovirus producer cells were transfected with 10 μg of MuSK wild‐type (WT) or mutant constructs and then grown for 72 hr in 7 ml of growth medium (Dulbecco's modified Eagle's medium [DMEM], 10% fetal calf serum [FCS], 1% prostate‐specific antigen [PSA]). A pBabe‐PURO‐EGFP empty vector was used as a control. The medium was harvested and centrifuged to remove cell debris, and stored at −80°C. C2C12 *Musk* KO cells were incubated with retrovirus overnight at 37°C and then replaced with growth medium. Cell selection with 1% puromycin was started 48 hr later and subsequently maintained in the growth medium for 7 days.

### 2.4 AChR clustering assay

C2C12 muscle cells were cultured in growth medium (DMEM, 15% FCS, 1% PSA) for 48 hr at 37°C until 90% confluency. Subsequently, the medium was changed to induce differentiation (DMEM, 2% FCS, 1% PSA), and cells were cultured for 5–7 days at 37°C until the formation of myotubes. Cells were incubated overnight (18 hr) with soluble full‐length human neuronal agrin for the induction of AChR clusters. Cells were incubated with Alexa Fluor® 594‐conjugated α‐bungarotoxin (cat no. #B13423; Life technologies) for 1 hr at 37°C, washed three times in phosphate‐buffered saline (PBS), and fixed for 10 min on 3% paraformaldehyde. Images were captured (with an operator blinded to the experiment) on IX71 Olympus fluorescent microscope using Simple PCI software (Digital Pixel Imaging Systems, Brighton, UK) and analyses using an automated macro for the ImageJ software (Figure S5,6).

### 2.5 Protein expression assays

Human *MUSK* cDNA (RefSeq NM_005592.3) was subcloned into pcDNA™3.1/Hygro(+) mammalian expression vector (cat no. #V87020; Invitrogen). *MUSK* mutations were introduced with QuikChange Multi Site‐Directed Mutagenesis Kit (cat no. #200514; Agilent) and confirmed by Sanger sequencing. For the analysis of MuSK expression, HEK‐293T cells were cotransfected with 3 μg of the constructs encoding WT or mutant MuSK and 100 ng of pEGFP‐N1 plasmid (to normalize for transfection efficiency) using polyethylenimine (PEI). Forty‐eight hours after transfection, the cells were resuspended in cold lysis buffer (10 mM Tris [pH 7.5], 100 mM NaCl, 1 mM, EDTA, 1% Triton X‐100, and protease inhibitor cocktail [cat no. #P8340; Sigma‐Aldrich]) and rotated for 1 hr at 4°C. Cell extracts were centrifuged for 15 min and resuspended into NuPAGE® lithium dodecyl sulfate (LDS) sample buffer (NP0007; Life Technologies) and NuPAGE® Sample Reducing Agent (NP0009; Life Technologies). Protein extracts were separated by sodium dodecyl sulfate‐polyacrylamide gel electrophoresis (SDS‐PAGE) electrophoresis and transferred onto the nitrocellulose membrane. The membrane was incubated with primary anti‐MuSK antibody (R&D, AF562) and secondary anti‐goat antibody conjugated to horseradish peroxidase (Dako). Detection was performed by using ECL (cat no. #RPN2106; GE Healthcare). For the analysis of MuSK localization, HEK‐293T cells were transfected with 3 μg of WT or mutant pMUSK‐mCherryN1 constructs using PEI, and fixed with 3% PFH 48 hr after transfection. 4′,6‐Diamidino‐2‐phenylindole (DAPI) was used for staining of the cell nuclei. Imaging was performed with a Zeiss LSM 880 confocal microscope. Protein stability studies were performed using cycloheximide at 20 μg/ml (cat no. #C7698; Sigma‐Aldrich).

### 2.6 Immunoprecipitation and coimmunoprecipitation assays

HEK‐293T cells were seeded at a density of 3 × 10^5^ cells per well in six‐well plates. The following day, cells were transfected with 3–6 µg of mammalian expression vectors encoding MuSK, DOK7‐EGFP, or LRP4‐EGFP. For immunoprecipitation from whole‐cell lysates, 48 hr after transfection cells were resuspended in 500 µl cold lysis buffer supplemented with protease inhibitor cocktail (cat no. #P8340; Sigma‐Aldrich) at 1:100 dilution and rotated for 1 hr at 4°C. Afterwards, the cell lysates were centrifuged for 15 min at 12000*g* at 4°C. A 20 µl aliquot of the supernatant was reserved as whole‐cell lysate loading control. Dynabeads® (Life technologies; cat no. #10003D) were resuspended and 50 µl per reaction was transferred to a tube. After washing three times in PBS, the beads were typically incubated with 5 µg of antibody at 4°C for 1 hr with gentle shaking. Then, the beads were added to the remaining supernatant and incubated at 4°C overnight with gentle rotation.

The beads were washed three times in 1 ml lysis buffer, resuspended in 25 µl of NuPAGE® LDS sample buffer 4X (Life Technologies, NP0007) with NuPAGE® Sample Reducing Agent 10X (NP0009; Life Technologies) and lysis buffer, and heated for 10 min at 90°C. For immunoprecipitation from the surface of HEK‐293T cells, the cells were incubated 48 hr after transfection with 5 µg of the selected antibody diluted in 500 µM culture medium at 37°C for 3 hr, washed three times in growth medium, and resuspended in 500 µl cold lysis buffer supplemented with protease inhibitor cocktail (cat no. #P8340; Sigma‐Aldrich) at 1:100 dilution.

Finally, the protein samples were separated by SDS‐PAGE electrophoresis, transferred onto nitrocellulose membrane, and analyzed by western blot analysis. For the detection of coimmunoprecipitated proteins, the nitrocellulose membrane was incubated with harsh stripping buffer (20 ml SDS 10%, 12.5 ml Tris HCl pH 6.8 0.5 M, 67.5 ml ultra‐pure water and 0.8 ml of β‐mercaptoethanol) and then reproved with the corresponding primary and secondary horseradish peroxidase (HRP)‐conjugated antibodies. Densitometry of bands was quantified with Image J software.

### 2.7 Phosphorylation assays

For the analysis of MuSK phosphorylation in HEK‐293T cells, 3 × 10^5^ cells were plated in six‐well plates and transfected with the constructs encoding WT or mutant MuSK and DOK7‐EGFP or LRP4‐EGFP if necessary. Forty‐eight‐hours after transfection, the cells were starved for 2 hr and then resuspended in cold lysis buffer supplemented with protease inhibitor (cat no. #P8340; Sigma‐Aldrich) and phosphatase inhibitor cocktails (cat no. #P8340; Sigma‐Aldrich). Immunoprecipitation and SDS‐PAGE electrophoresis were performed as previously described.

The membrane was blocked in 5% bovine serum albumin at 4°C overnight. For the detection of MuSK tyrosine phosphorylation, the membrane was incubated with 1:1000 anti‐phosphotyrosine antibody clone 4G10® (cat no. #16‐101; Millipore) and secondary HRP‐conjugated goat anti‐mouse antibody (P0447; Dako). For the detection of MuSK, the membrane was incubated with harsh stripping buffer (20 ml SDS 10%, 12.5 ml Tris HCl pH 6.8 0.5 M, 67.5 ml ultra‐pure water and 0.8 ml of β‐mercaptoethanol) and then reproved with anti‐MuSK antibody (cat no. #AF562; R&D) at 1:300 dilution and secondary HRP‐conjugated rabbit anti‐goat antibody (P0449; Dako). Detection was performed by using ECL (cat no. #RPN2106; GE Healthcare).

For the analysis of MuSK phosphorylation in muscle cells, C2C12 *Musk* KO muscle cells overexpressing MuSK (RefSeq NM_005592.3) WT or mutant variants were differentiated at 37°C for 5–7 days until the formation of myotubes. The cells were starved for 2 hr and subsequently incubated with full‐length human agrin at 1:100 dilution. Cells were harvested at 0 min, 10 min, 40 min, 60 min, 2 hr, 4 hr, 12 hr, and 24 hr after agrin incubation, and protein extracts were processed as previously described in this section. Special care was taken to assure that the beads‐protein incubation time was similar across samples. MuSK tyrosine phosphorylation was detected as previously described. The study of MuSK phospho‐serine 751 was conducted using previously described antibodies against S751 (Camurdanoglu et al., 2016).

### 2.8 Molecular modeling of MuSK structure

Modeling of MuSK variants was performed with PyMOL v1.7.4.5 Edu. A proposed model of MuSK KD structure upon dimerization was generated by substitution of the epidermal growth factor receptor (EGFR) KD dimer (Zhang, Gureasko, Shen, Cole, & Kuriyan, 2006) and the insulin‐like growth factor 1 receptor (IGF1R) KD dimer (Cabail et al., 2015) with two copies of the MuSK KD.

## 3 RESULTS

### 3.1 Clinical features

This female patient had onset of symptoms at birth with hypotonia, severe muscle weakness, respiratory problems, and failure to thrive. She underwent tracheostomy soon after birth due to congenital upper airways obstruction secondary to bilateral vocal cord palsy. She was PEG‐fed from an early age. Electromyography showed abnormal repetitive nerve stimulation (RNS) of *anconeus* and deltoid muscles (>20% decrement) and abnormal single fiber electromyography of *extensor digitorum communis* (mean consecutive difference: 65.8 µs; normal: 30 µs) compatible with a disorder of neuromuscular transmission. Of note, RNS of distal muscle *abductor digiti minimi* was normal. AChR and MuSK antibodies were absent. At the age of 2 years, she was started on pyridostigmine at 4 mg·^−1^kg·^−1^day. After initial improvement over the first 48 hr, there was a severe deterioration in muscle strength with the need for ventilation and intensive care support during the following 4 weeks. After discharge, the patient was maintained off‐treatment and confined to a wheelchair until the age of 18 years when she was seen at the Oxford Referral Center for CMS (John Radcliffe Hospital, Oxford, UK). Examination at that time showed fatigable ptosis, mild facial weakness and normal eye movements. She was PEG‐fed and had a permanent tracheostomy. Muscle strength was profoundly decreased proximally in upper and lower limbs with inability to produce movements against gravity (medical research council [MRC] scale 2/5). Muscle strength in distal muscles was less severely impaired (MRC scale 4/5). The patient was unable to walk, and only able to stand for few seconds with bilateral aid. She required continuous use of a wheelchair and was dependent for most activities of daily living. Her Quantitative Myasthenia Gravis score (severity) was 29 out of 39 (Jaretzki et al., 2000). A patient muscle biopsy was not available to study the endplate structure.

### 3.2 Identification of MUSK mutations

Initial screening for mutations in *CHRNE, COLQ, DOK7*, and *RAPSN* by bidirectional sequencing of PCR amplicons was negative. The sequencing of the coding exons and flanking regions of *MUSK* revealed two missense variants in *MUSK* (RefSeq NM_005592.3; Figure 2a). The first variant was a transition in exon 9, leading to the substitution of a positively charged arginine replacing one of the cysteine residues in the frizzled‐like cysteine‐rich domain (c.949T>C; p.Cys317Arg). The second variant was a transition in exon 14, leading to the substitution of alanine to valine in the kinase domain (c.1850C>T; p.Ala617Val). Both the Cys317 and Ala617 residues are conserved across species (Figure 2b). Sanger sequencing showed segregation of both *MUSK* variants within the family (Figure 2c**)**. Neither of the variants are listed in Ensembl genome browser 94 (EMBL‐EBI, Cambridge, UK; URL: https://www.ensembl.org; March 2019; Zerbino et al., 2018) or in the 125,748 exome sequences and 15,708 whole‐genome sequences from unrelated individuals of the Genome Aggregation Database (gnomAD, Cambridge, MA; URL: http://gnomad.broadinsitute.org; March 2019; Lek et al., 2016). In silico analysis classified both variants as probably damaging by Mutation Taster 2 and PolyPhen‐2 (Table S1). The results from a more comprehensive analysis using dbNSFP 4.0 are included as supplementary data. Both variants have been submitted to an online public database (https://www.ncbi.nlm.nih.gov/clinvar/)

### 3.3 Molecular modeling of MuSK variants

**Figure 3:**
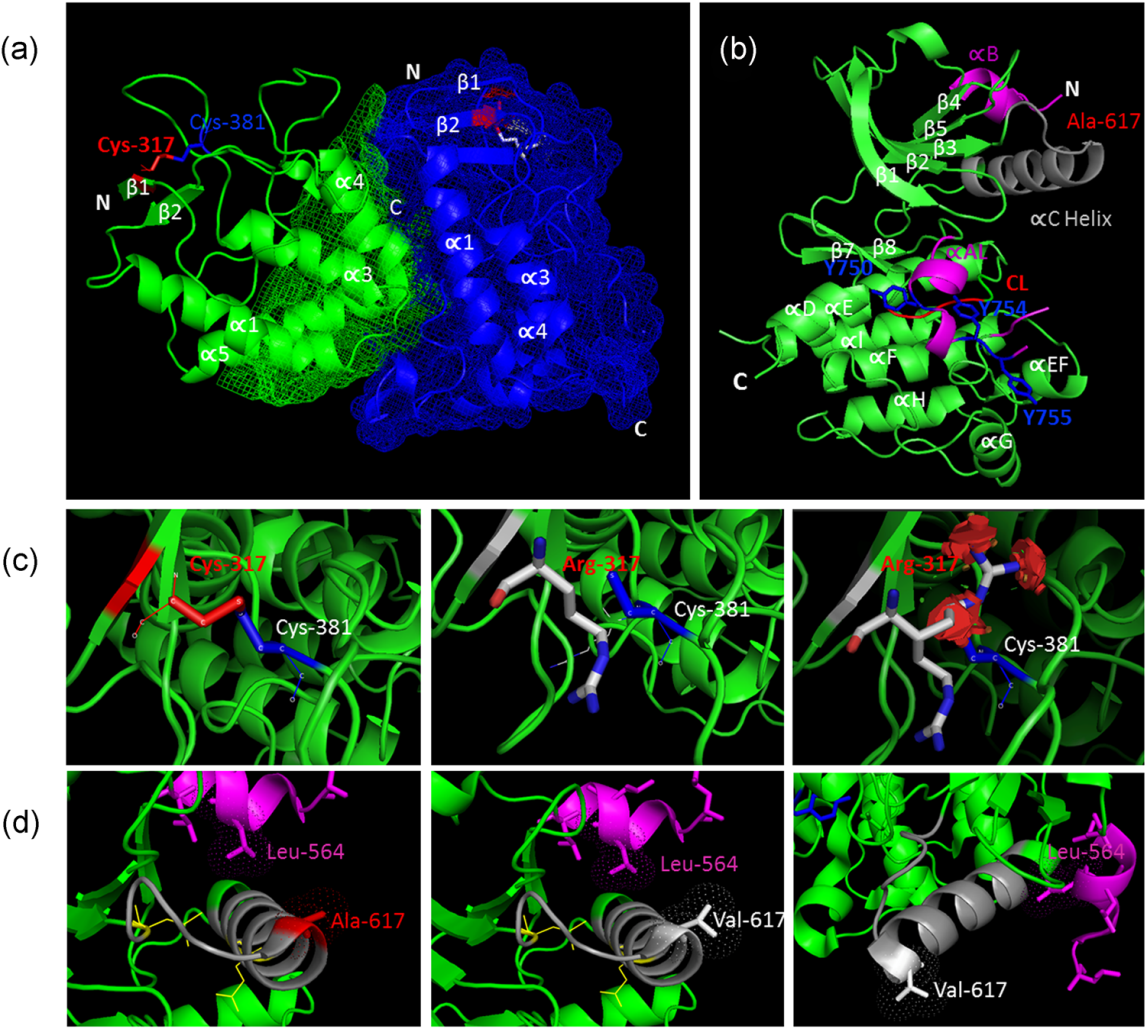
Molecular modeling of MuSK variants. (a) Cartoon representation of MuSK Frizzled‐like domain structure (MMDB ID: 76272) with two copies of the asymmetric dimmer colored in green and blue. Structural elements are labeled in white text according to (Stiegler, Burden, & Hubbard, 2009) and the p.Cys317 residue in red text. (b) Cartoon representation of MuSK KD monomer structure (MMDB ID: 20673). Structural elements are labeled according to (Till et al., 2002). MuSK KD structure is made of β‐strands and α‐helices (green). MuSK activity is regulated by the juxtamembrane domain at the N‐terminus (αB) and the activation loop (αAL; pink). The catalytic loop (CL) is represented in red color. The αC‐helix is colored gray and the p.Ala617 residue is labeled in red. The tyrosine residues within the activation loop (Tyr‐750/754/755) are shown in stick representation (blue). The crystal structure of the MuSK KD after dimerization is not known. (c) The residue p.Cys317 forms a disulfide bond with p.Cys381. Modeling of p.C317R shows how the substitution of cysteine to an arginine result in the disruption of an essential disulfide bond leading to multiple clashes with the neighboring residues. (d) The p.Ala617 residue (red color) is located in the αC‐helix at the N‐terminal lobe of the MuSK KD. Using the crystal structure by (Till et al., 2002), the substitution of alanine to valine does not result in changes in distance and/or a clash with other residues. KD, kinase domain; MuSK, muscle‐specific receptor tyrosine kinase

The p.Cys317 residue contributes to the formation of an essential disulfide bond for MuSK FzD architecture (Figure 3a). The p.Ala617 residue plays a part in the formation of the αC‐helix in the N‐terminal lobe of MuSK KD (Figure 3b), which may be necessary for the optimal positioning of critical lysine residues with respect to ATP binding (Till et al., 2002). The p.Cys317Arg substitution results in disruption of an essential disulfide bond (Figure 3c). The p.Ala617Val substitution does not cause changes in distance or clash with other residues using the crystal structure currently available for the MuSK KD monomer (Figure 3d). The structure of the MuSK KD dimer has not yet been resolved (Till et al., 2002). Understanding the pathogenic mechanisms of the MuSK p.Ala617Val substitution at the structural level based on the monomer structure is challenging because the αC‐helix from the kinase domain is exposed to a large void in the crystal (Till et al., 2002).

### 3.4 Effects of MuSK variants p.Cys317Arg and p.Ala617Val on AChR clusters formation

**Figure 4:**
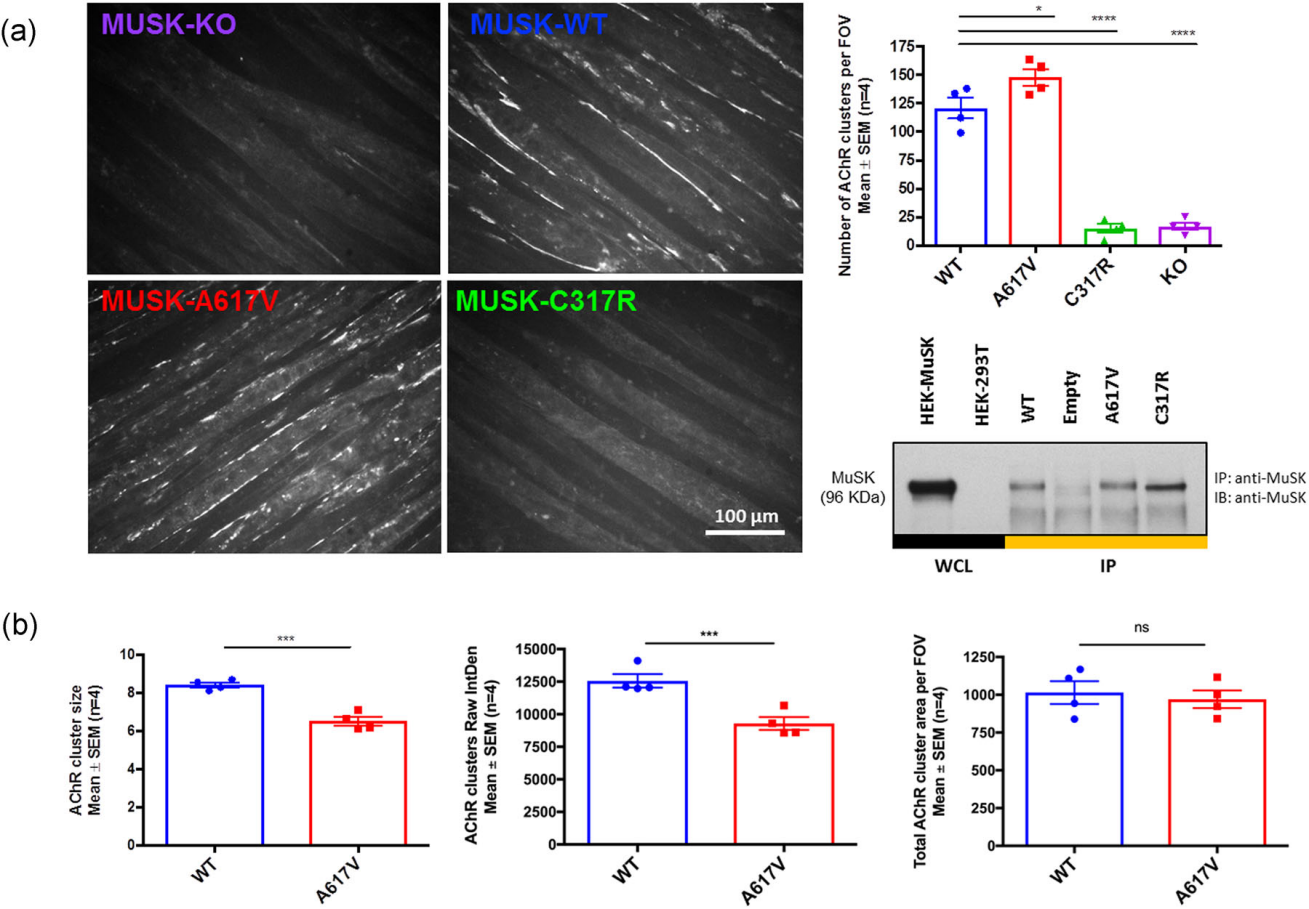
Effect of MuSK variants on AChR clustering. (a) The retroviral infection of C2C12 *MusK* KO recovered successfully MuSK protein expression and AChR clustering function. Bar graphs show the quantification of AChR clusters from MuSK WT and variants. Data points represent the mean data from 40 images for each condition per experiment (*n* = 4). One‐way ANOVA and Dunnet's multiple comparisons test (*p < .05; ***p < .001). (b) Quantification of average AChR cluster size, total AChR cluster area per field of vision (FOV), and raw integrated density in MuSK WT and p.A617V myotubes. Data points represent the mean data from 40 images for each condition per experiment (*n* = 4). Two‐tailed unpaired *t* test (***p < .01). ANOVA, analysis of variance; KO, knock‐out; MuSK, muscle‐specific receptor tyrosine kinase; WT, wild‐type

*Musk* KO C2C12 mouse muscle cells were generated with CRISPR‐Cas9 nickase genome‐editing technology via non‐homologous end‐joining (Methods section). Retroviral infection of C2C12 *Musk* KO cells recovered protein expression and AChR clustering function (Figure 4a). *Musk* KO cells infected with an empty vector were used as a negative control. The p.Cys317Arg variant abrogated AChR clustering by reducing the number of AChR clusters (greater than 2.0 μm in length) by more than 90% compared with WT (*p* < .0001), while the p.A617V variant caused a statistically significant increase of approximately 23% (*p* = .029). The analysis of the average AChR cluster size showed that the p.A617V clusters were approximately 23% smaller in size (*p* < .001; Figure 4b) while the total AChR cluster area remained unchanged (*p* = .159). Overall, these results show that the MuSK p.A617V mutation underlies abnormalities in the clustering process that causes the AChR clusters to appear fragmented. In addition, the analysis of RawIntDent (sum of pixel values from each AChR cluster) showed that AChR clusters from p.A617V myotubes were 26% less bright compared with WT (*p* < .001). Comprehensive images from MuSK WT and mutant myotubes are also provided (Figures S7–9).

### 3.5 Effect of MuSK variants p.Cys317Arg and p.Ala617Val on expression, localization, and protein interaction

**Figure 5:**
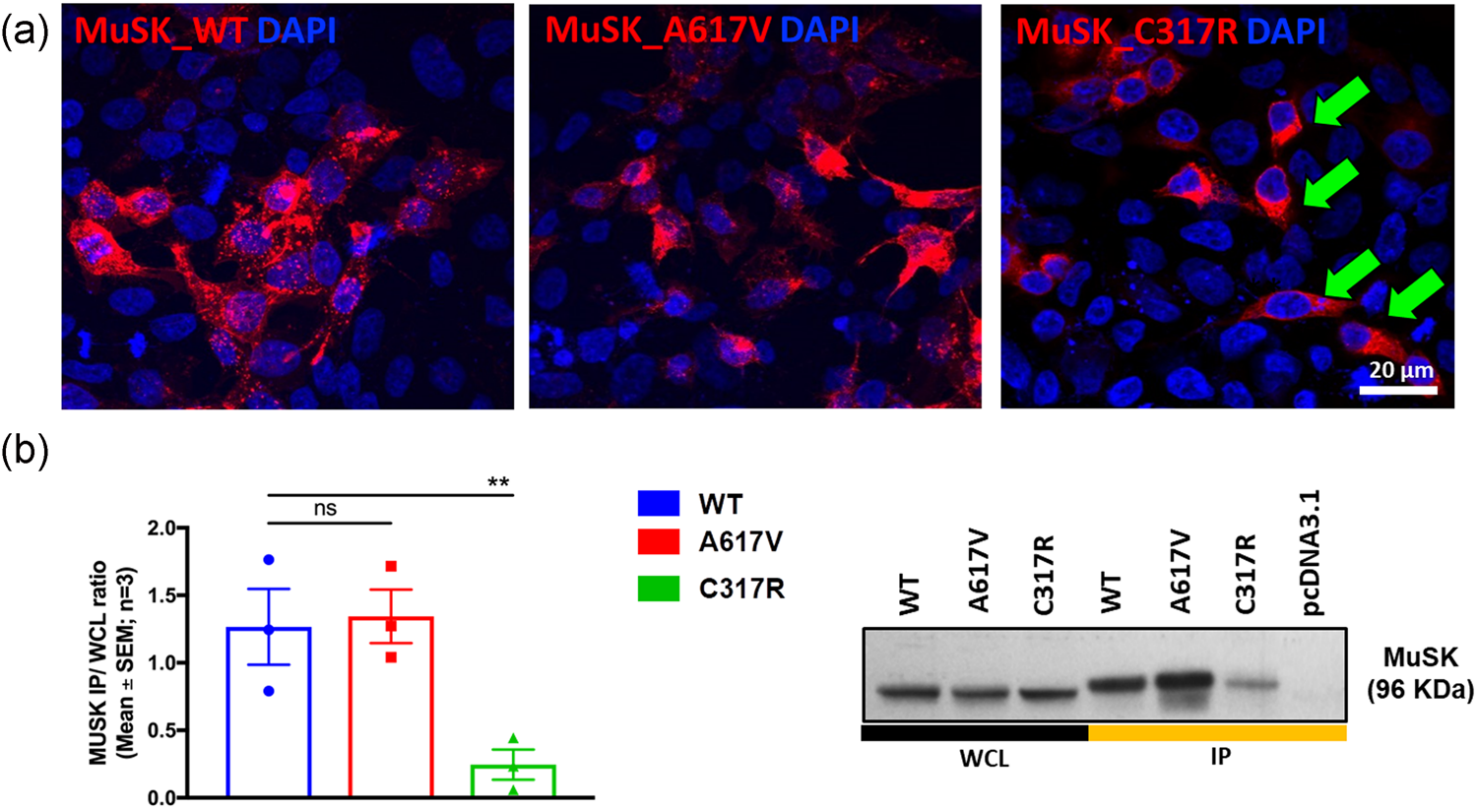
Effect of MuSK variants on protein expression, stability, and localization. (a) MuSK WT and p.A617V expressed correctly at the cell surface of HEK‐293T cells after PEI transfection of WT and mutant pMUSK‐mCherryN1 constructs, while MuSK p.C317R remained mostly cytoplasmic (green arrows). Cell nuclei were stained with DAPI. (b) Pull‐down of MuSK p.C317R from the cell surface of HEK‐293T was reduced to 19.43 ± 8.78% compared with WT. The results (ratio IP: WCL) are represented as mean ± *SEM* from *n* = 3 experiments. One‐way ANOVA and Dunnett's multiple comparisons test (**p < .01). ANOVA, analysis of variance; DAPI, 4′,6‐diamidino‐2‐phenylindole; IP, immunoprecipitation; PEI, polyethylenimine; MuSK, muscle‐specific receptor tyrosine kinase; *SEM*, standard error of mean; WCL, whole cell lysates; WT, wild‐type

To investigate whether the functional effects of MuSK variants were related to abnormalities in protein expression, human mCherry‐tagged MuSK expression constructs were expressed in HEK‐293T cells and visualized under fluorescence microscopy. This showed that MuSK p.C317R insertion into the cell surface was severely compromised with the majority of the mutant protein remaining cytoplasmic (Figure 5a; green arrows). Pull‐down assays from the cell surface showing a dramatic reduction in the amount of MuSK immunoprecipitated confirmed this result (Figure 5b). It is likely that the loss of the cysteine, key to the structure of the FzD, causes misfolding of the protein resulting in severely reduced cell surface expression. No abnormalities were seen for the p.A617V variant. The small shift observed in protein mobility between immunoprecipitation (IP) samples and whole‐cell lysates may be related to differences in salt concentration derived from the use of IP buffers. A cycloheximide chase assay over a 24 hr incubation period showed similar stability for MuSK WT and p.A617V proteins (Figure S10).

Coimmunoprecipitation assays from the surface of transiently transfected HEK‐293T cells were used to address whether MuSK p.A617V was impairing the interaction with other partners of the LRP4‐MuSK‐DOK7 complex. MuSK p.A617V had no major effect on the amount of LRP4 or DOK7 being pulled‐down, indicating normal protein interaction (Figure S11).

### 3.6 Effect of MuSK p.Ala617Val on MuSK phosphorylation

**Figure 6:**
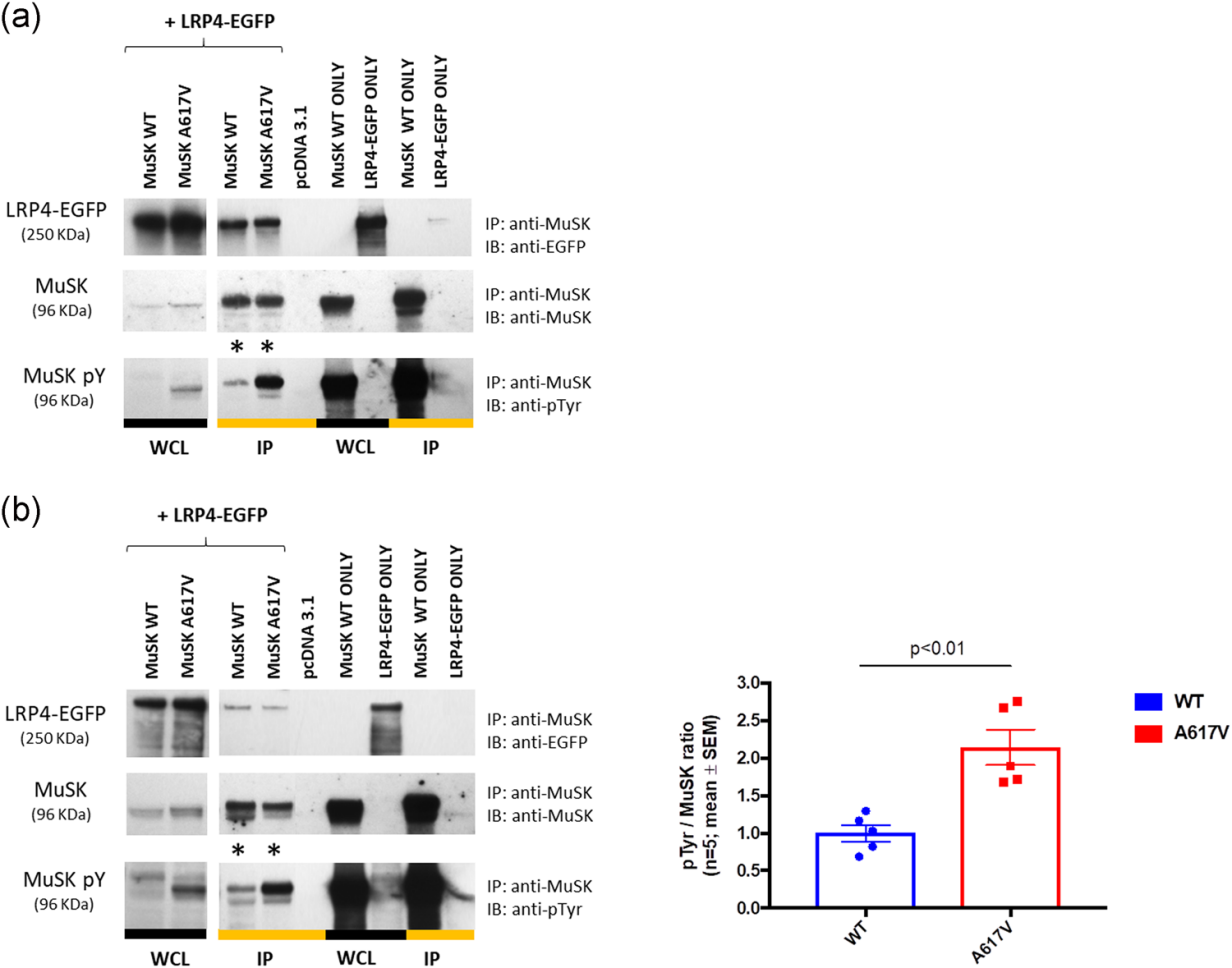
Effect of p.A617V on MuSK tyrosine phosphorylation in HEK‐293T cells. (a) MuSK tyrosine phosphorylation was increased in the p.A617V variant (*) compared with WT following incubation with full‐length agrin for one hour. (b) MuSK tyrosine phosphorylation was also significantly increased in the p.A617V variant (*) in the absence of agrin incubation as shown by the densitometry analysis. Results are shown as mean ± *SEM* relative to WT from *n* = 5 experiments. Two‐tailed unpaired *t* test (*p* < .01). Lanes 1 and 2 are loading controls while lanes 5–9 are controls to ensure the specificity of the pull‐down assay. EGFP, enhanced green fluorescent protein; IP, immunoprecipitation; MuSK, muscle‐specific receptor tyrosine kinase; *SEM*, standard error of mean; WCL, whole cell lysates; WT, wild‐type

First, we investigated if the p.A617V mutation was affecting MuSK tyrosine phosphorylation using HEK‐293T cells as a standard heterologous expression system. Next, we used C2C12 mouse muscle cells as a more physiological system that naturally incorporates the constituents of muscle AChR clustering and dispersal systems. Coimmunoprecipitation experiments in HEK‐293T cells overexpressing MuSK and LRP4 following incubation with full‐length agrin for 1 hr, showed that MuSK tyrosine phosphorylation was increased by approximately twofolds in the p.A617V variant compared with WT (Figure 6a). The increase in phosphorylation was also seen independently of agrin incubation (*p* < .01; *n* = 5; Figure 6b), and thus MuSK p.A617V was found to be constitutively activated in the presence of LRP4. No significant differences in tyrosine phosphorylation were observed when MuSK was either expressed alone or coexpressed with DOK7 (Figure S12). These results suggested that p.A617V affects MuSK tyrosine phosphorylation when interacting with LRP4.

**Figure 7:**
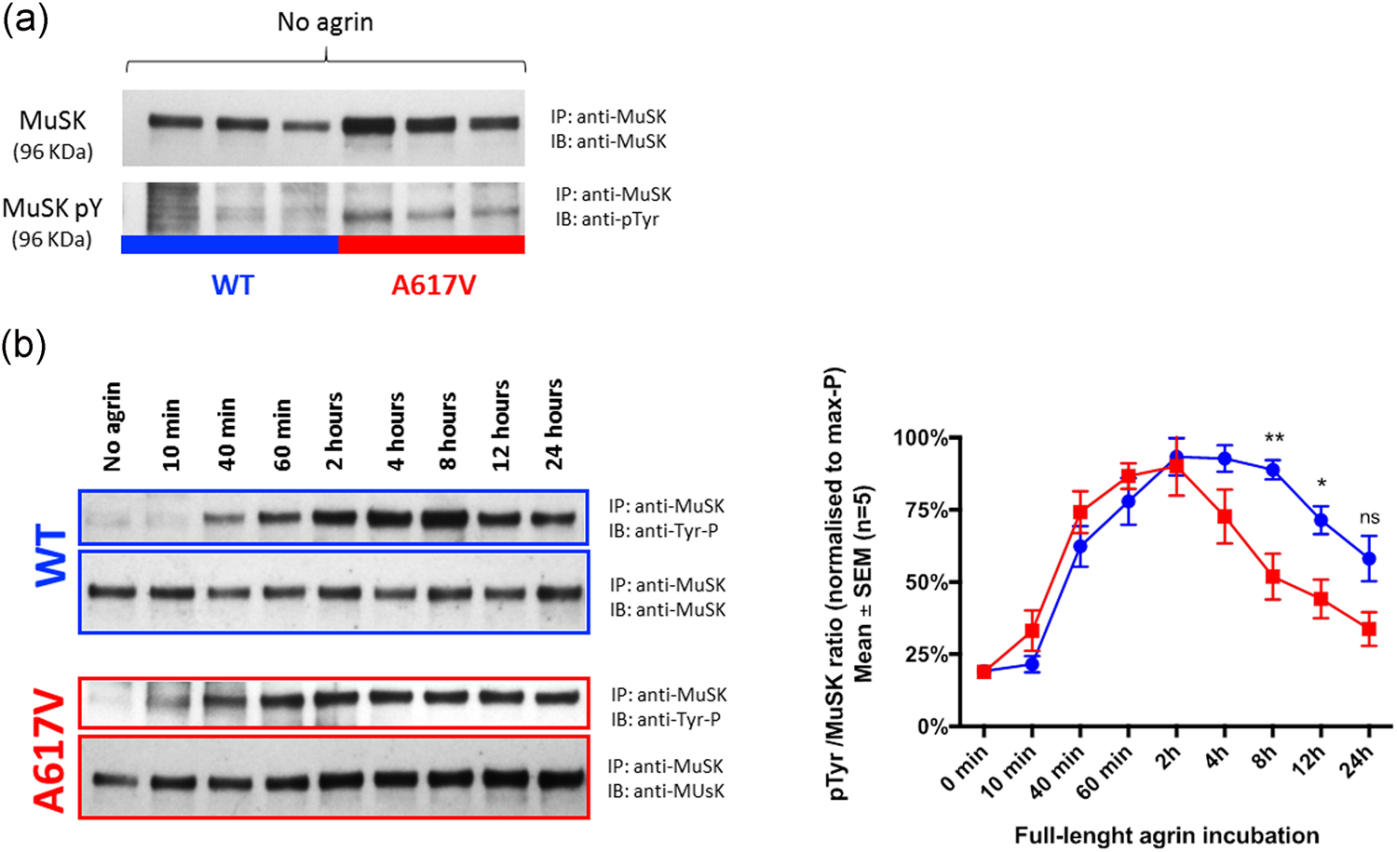
Effect of p.A617V on baseline MuSK kinase activity and agrin‐induced temporal activation profile in muscle cells. (a) In the absence of agrin, there was increased phosphorylation signal in the p.A617V variant compared with WT (*n* = 3). (b) Analysis of the temporal activation profile of MuSK over a 24‐hr period following incubation with full‐length human agrin. The pTyr/MuSK ratio from the different timepoints was calculated for each individual experiment relative to its maximum phosphorylation ratio (100%). The results are shown as mean ± *SEM* from *n* = 5 experiments. Repeated measures two‐way ANOVA (*p* = .003). Representative images of western blots are provided. IB, immunobinding; IP, immunoprecipitation; MuSK, muscle‐specific receptor tyrosine kinase; *SEM*, standard error of mean; WT, wild‐type

MuSK tyrosine phosphorylation was studied in C2C12 *Musk* KO myotubes infected with retrovirus expressing human MuSK (RefSeq NM_005592.3) WT or mutant constructs in the absence and presence of full‐length agrin. In the absence of agrin incubation, there was an increased tyrosine phosphorylation signal in the p.A617V variant compared with WT (Figure 7a). In keeping with the previous observations in HEK‐293T cells, MuSK p.A617V was found to be constitutively activated in muscle cells. The analysis of the temporal activation profile of MuSK over a 24‐hr period following incubation with full‐length agrin showed significant differences in the pattern of MuSK tyrosine phosphorylation between WT and p.A617V myotubes (*p* = .003; Figure 7b). An initial faster increase in MuSK p.A617V kinase activity after incubation with agrin was observed followed by a deeper decrease from 2 hr onwards. Agrin‐induced MuSK tyrosine phosphorylation was nullified in MuSK p.C317R myotubes in agreement with the lack of agrin‐induced AChR clusters previously shown (Figure S13).

### 3.7 Hypothesis of the potential molecular mechanisms leading to abnormal levels of MuSK phosphorylation

#### 3.7.1 Dysregulation of MuSK kinase activity

A serine phosphorylation site (S751) in the kinase loop of MuSK has been recently proposed to contribute to the modulation of MuSK kinase activity (Camurdanoglu et al., 2016). Immunoblotting with an antibody specific for detection of MuSK S751 phosphorylation levels did not show significant differences between WT and p.A617V myotubes after incubation with full‐length agrin (Figure S14). In addition, S751 phosphorylation levels showed no differences between MuSK WT and p.A617V in HEK‐239T cells. These results show that the abnormalities seen in tyrosine phosphorylation derived from the p.A617V substitution are not caused by differences in the S751‐related modulation of MuSK activity.

#### 3.7.2 Abnormal dimerization of MuSK kinase domains

Insights from studies done on other receptor tyrosine kinases (RTKs) have shown that dimerization of kinase domains can occur in symmetric and asymmetric ways (Lemmon & Schlessinger, 2010; Zhang et al., 2006). Based on the fact that there are many conserved features among kinases regarding their structures and catalytic mechanisms (Hanks, Quinn & Hunter, 1988), a model of MuSK‐KD dimerization was generated by homology modeling based on the KD dimer crystal structures of two well‐known RTKs: IGF1R and EGFR.

**Figure 8:**
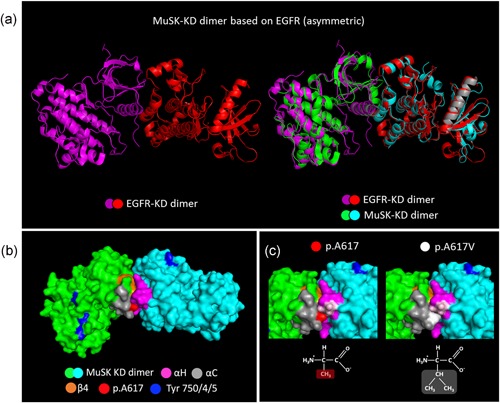
Molecular modeling of MuSK KD dimerization based on the EGFR‐KD dimer structure. (a) MuSK KD asymmetric dimer built by homology modeling based on the crystal structure of the EGFR‐KD activated dimer (MMDB ID: 40024). (b) Surface representation of MuSK KD dimer (green and cyan) based on the EGFR dimer. In the proposed structure, MuSK KDs, colored green and cyan, form an asymmetric dimer with the H‐helix (pink) of one monomer binding to αC‐helix (gray) and β4 sheet (orange) from the contralateral monomer. The p.Ala617 residue (red) is located within the αC‐helix at the dimerization interface. The tyrosines within the activation loop (Tyr‐750/754/755) are in blue. (c) The figure shows how the p.Ala617Val substitution modifies the dimerization interface increasing the hydrophobic interface for the docking of the contralateral MuSK KD. In this model, the p.Ala617 residue (red) interacts with the p.Ala842 and p.Asp843 residues (light purple) from the H‐helix (purple) at the dimerization interface. EGFR, epidermal growth factor receptor; KD, kinase domain; MuSK, muscle‐specific receptor tyrosine kinase

IGF1R‐KDs form a symmetric dimer where the juxtamembrane region of one monomer interacts with the αC‐helix of the contralateral monomer (Cabail et al., 2015). In the three‐dimensional sequence alignment of MuSK‐KD with IGF1R‐KD, MuSK p.Ala617 residues are exposed to a void in the crystal and there are no obvious effects on the structure derived from the p.A617V substitution (Figure S15). By contrast, the EGFR‐KDs form an asymmetric dimer (Figure 8a) essential for EGFR activation where the αH‐helix of one monomer interacts with αC‐helix and β4 sheet of the contralateral monomer, and the kinase core is dominated by hydrophobic interactions (Zhang et al., 2006). The three‐dimensional sequence alignment of MuSK‐KD with EGFR‐KD locates the MuSK p.Ala617 residue at the αC‐helix, which is part of the dimerization interface and responsible for the interaction of the two monomers (Figure 8b). As represented, the p.A617V substitution at the αC‐helix could increase the hydrophobic interface for the docking of the contralateral monomer resulting in a more stable dimer with increased catalytic activity (Figure 8c).

### 3.8 Response to treatment with β2‐adrenergic agonists

Based on the previous experience from our group with β2‐adrenergic agonists in patients with *DOK7* mutations (Lashley, Palace, Jayawant, Robb, & Beeson, 2010), treatment with oral salbutamol 4 mg twice a day (0.2 mg/kg/day) was initiated. The drug had a remarkable effect on muscle strength and functionality from the first month onwards. From being unable to walk, after 6 months the patient was able to walk short distances and performed the 10‐meter timed walk test unaided in 25 s (Figure S16). Her leg rise time at 4°C increased from 0 to 34 s. This had a definitive impact in the quality of life and activities of daily living, as measured by the Barthel activities of daily live index (Mahoney & Barthel, 1965). The beneficial effect has persisted over the 24‐month follow‐up period since the initiation of treatment.

## 4 DISCUSSION

MuSK is considered the key organizer of NMJ formation and maintenance and is an essential element of the agrin‐LRP4‐MuSK‐DOK7 signaling pathway (Burden et al., 2013; DeChiara et al., 1996). We explore the mechanisms underlying two novel missense variants in *MUSK* causing CMS. The findings point to a novel pathogenic mechanism highlighting that constitutive MuSK phosphorylation can be detrimental in the pathway that governs synaptic structure. The study was also useful in directing appropriate pharmacological therapy to the patient with β2‐adrenergic agonists, and the clear response to salbutamol provides further support for pathogenicity of the p.A617V substitution.

The p.C317R mutation ablated AChR clustering and MuSK phosphorylation after agrin incubation, thus behaving as a null mutation. The p.Cys317 residue contributes to the formation of an essential disulfide bond for MuSK FzD architecture, which is disrupted by the cysteine to arginine substitution. Although MuSK FzD is dispensable for synapse formation and maturation (Remédio et al., 2016), the impairment of the FzD architecture results in a dramatic reduction of MuSK cell surface expression, which cannot then be activated by its extracellular ligand agrin. Only one mutation (P344R) has been reported in this domain to date although functional studies were not conducted (Mihaylova et al., 2009).

In cell culture, the p.A617V mutation caused AChR clusters to appear fragmented and altered MuSK phosphorylation. The clustering abnormalities seen in p.A617V myotubes were demonstrated through the presence of a higher number of individual AChR aggregates, which were significantly smaller in size and also had reduced fluorescence intensity compared with WT myotubes. No differences were observed in the total AChR cluster area of p.A617V myotubes compared with WT. The constitutive activation of MuSK in HEK‐293T cells and the time‐dependent reduction in MuSK tyrosine phosphorylation levels in muscle cells following agrin activation suggest that precise MuSK phosphorylation levels are crucial to maintain the integrity of AChR clusters. p.A617V reveals that disrupting the control of MuSK phosphorylation levels may well be a newly identified pathogenic mechanism underlying NMJ disorders.

MuSK activation is closely related to its dimerization (Hopf & Hoch, 1998). To understand how MuSK A617V is constitutively activated and how this leads to the reduction of agrin‐induced MuSK phosphorylation levels is challenging in the absence of a crystal structure for the MuSK KD dimer. Insights from the study of other protein kinases have shown that the dimerization of KDs can occur in symmetric or asymmetric ways (Zhang et al., 2006). It is important to note that the dimerization of the extracellular domain of RTKs that follows the binding of extracellular ligands is a separate process from the subsequent dimerization of the intracellular KD domain and transphosphorylation of tyrosine residues (Lemmon & Schlessinger, 2010). The 3D model of MuSK KD asymmetric dimer based on EGFR predicts that the p.Ala617 residue is located at the dimerization interface and that the p.A617V substitution could increase the hydrophobic interface for the docking of the contralateral monomer resulting in a more stable dimer. A previous study showed elevated basal levels of MuSK phosphorylation in HEK‐293T cells in the p.M835V mutation (Ammar et al., 2013), implying that there could be a common pathogenic mechanism for p.A617V and p.M835V mutations. The location of the p.Met835 residue in the asymmetric model is also predicted to be within the dimerization interface in close interaction with p.Leu560 at the juxtamembrane domain from the contralateral monomer (Figure S17) providing support for this pathogenic model. Of note, it has been previously suggested that p.Leu560 may be a site for protein–protein interactions (Till et al., 2002).

Other potential pathogenic mechanisms at the molecular level may include changes in the allosteric conformational equilibrium derived from the amino acid substitutions (Weinkam, Chen, Pons, & Sali, 2013) leading to loss of kinase autoinhibition by the juxtamembrane domain or the activation loop. Another possibility would be that the p.A617V substitution is affecting the dynamics of the αC‐helix resulting in reduced autoinhibition (Dixit & Verkhivker, 2011; Palmieri & Rastelli, 2013).

The constitutive activation of MuSK caused a reduction in agrin‐induced MuSK tyrosine phosphorylation levels in p.A617V myotubes over time, indicating the existence of regulatory mechanisms not present in HEK‐293T cells. It is believed that MuSK activation levels are set, at least in part, by tyrosine phosphatases, and that MuSK signaling normally activates tyrosine phosphatases to limit its own spread (Madhavan & Peng, 2005). This fine balance of MuSK stimulation with that of tyrosine phosphatases could modulate the level, duration or range of the MuSK signaling, which would help cluster AChRs at sites where MuSK is maximally activated. It is also known that this balance is important for the maintenance and stabilization of AChR clusters (Camilleri et al., 2007) in keeping with tyrosine kinase inhibitors dispersing agrin‐induced preformed AChR clusters (Ferns, Deiner, & Hall, 1996). It may be that the constitutive activation of p.A617V is impairing agrin‐induced MuSK tyrosine phosphorylation levels by affecting the tight coupling between kinase and phosphatase activities that is essential for the formation and maintenance of AChR clusters.

*MUSK* mutations are rare with only a few cases reported to date (Engel, Shen, Selcen, & Sine, 2015). This differs from the much wider spectrum of *DOK7* mutations (Cossins et al., 2012), which account for approximately a fifth of the CMS population in the United Kingdom (Finlayson, 2013). The underlying reason explaining this phenomenon is unknown but could be related to the fact that severe *MUSK* mutations are incompatible with life due to the crucial role of MuSK as the key organizer at the postsynaptic membrane. The clinical phenotype reported in individuals with *MUSK* mutations is variable and differs in both severity and time of onset (Chevessier et al., 2004; Gallenmüller et al., 2014; Giarrana et al., 2015; Luan et al., 2016; Maselli et al., 2010; Mihaylova et al., 2009; Murali et al., 2019; Owen et al., 2018). The current case lies on the severe side of the clinical spectrum with the neonatal onset and life‐threatening complications. Overall, there are clear phenotypic similarities between MuSK and DOK7‐CMS patients (Palace et al., 2007).

The first CMS case described with *MUSK* mutations was reported to have a positive response to treatment with pyridostigmine 120–240 mg and 3,4‐DAP 30–50 mg daily (Chevessier et al., 2004). However, subsequent reports have shown that cholinesterase inhibitors were not beneficial in other cases and 3,4‐DAP was only partially effective. By contrast, treatment with β2‐adrenergic agonists resulted in significant improvement in several cases reported (Gallenmüller et al., 2014; Maselli et al., 2010; Owen et al., 2018). There is increasing evidence to suggest that β2‐adrenergic agonists stabilize motor endplates' structure and compensate for the impairment in the agrin‐LRP4‐MuSK‐DOK7 signaling pathway (Clausen, Cossins, & Beeson, 2018; Mcmacken et al., 2018). A recent study has proposed that NMJs are innervated by sympathetic neurons (Khan et al., 2016) suggesting that this could be the target of β2‐adrenergic agonists at the NMJ.

In conclusion, this study shows that MuSK p.C317R and p.A617V are pathogenic mutations responsible for the patient's myasthenic syndrome and uncovers that levels of MuSK phosphorylation are critical to maintain the synaptic structure. Our study also highlights the importance of understanding disease mechanisms to guide appropriate pharmacological treatment.

## Supporting information

Supporting information

Supporting information

## Data Availability

The data that support the findings of this study are available from the corresponding author upon reasonable request. Both variants reported have been submitted to an online public database (https://www.ncbi.nlm.nih.gov/clinvar/).
