# Supplementary material for: Congenital myasthenic syndrome due to mutations in MUSK suggests that the level of MuSK phosphorylation is crucial for governing synaptic structure
Source: Hum Mutat. 2019 Nov 25;41(3):619–31. doi: 10.1002/humu.23949 (PMC7028094; doi:10.1002/humu.23949)
Supplement: Supplementary file 2 — Supporting information [file HUMU-41-619-s002.docx]

**SUPPLEMENTARY INFORMATION**

## Generation of C2C12 MuSK KO cell lines

### Use of CRISPR-Cas9 genome engineering system

C2C12 myoblasts were electroporated with 10μg of each pX335_Cas9n plasmid containing the single strand oligonucleotides MUSK guides A or B in order to produce spontaneous indels via non-homologous end-joining leading to protein truncation. Myoblasts from different clones were differentiated into myotubes by serum starvation and incubated with full-length agrin overnight for the induction of AChR clusters. From the 40 starting clones, 10 (25%) managed to differentiate into myotubes and 4 (10%) had complete absence of AChR clusters after incubation with agrin. A number of clones producing abnormal clusters were considered non-optimal **(Figure S1).** The clonal line found to readily differentiate into myotubes (KO-1) was selected for further experiments.

The DNA from this clone was extracted, amplified by PCR of genomic DNA and resolved by gel electrophoresis. Sanger sequencing of PCR products was difficult to interpret due to the presence of multiple overlapping sequences. Therefore, the PCR products were subcloned into pGEM-T-Easy for segregation. Sanger sequencing analysis of PCR products after segregation showed the presence of 4 different DNA sequences **(Figure S2)**. This suggests that C2C12 is very likely to be a polyploid cell line, which contains more than 2 copies of *Musk* in a single cell. One study looking at the morpho-functional characterisation of C2C12 has also proposed this idea (Burattini et al., 2004). An alternative, but less likely possibility, would be the generation of new indels by the Cas9n after clonal isolation

Figure S1. Representative images of AChR clusters in C2C12 myotubes engineered with CRISPR-Cas9n after incubation with agrin. Representative microscopic fields of AChR clusters labelled with Alexa**®** Fluor 594**-**α-bungarotoxin showing the variety of AChR clusters resulting from the gene-editing process. Clonal lines producing normal **(A)** or abnormal **(B)** clusters were discarded as not indicative of definitive *Musk* KO. Only clonal lines completely lacking AChR clusters after agrin incubation were selected as appropriate **(C)**.

Figure S2. Genotype analysis of CRISPR-generated *Musk* KO clonal line

**(A)** Genomic PCR analysis of the *Musk* region comprising the CRISPR target sites for WT and different *Musk* KO clonal cell lines. Expected product size was 810 bp in size.

**(B)** The PCR products from genomic DNA of KO-1 were subcloned into pGEM-T-Easy and segregated. Ten of the purified plasmids were analysed by DNA sequencing and 4 different variants of *Musk* compared to Ref Seq NM_001037127.2 were identified:

i) Representation of *Musk* exon 3 and genomic boundaries (Ref Seq NM_001037127.2) including protospacer adjacent motifs (PAM) and genomic targets of CRISPR Cas9 gRNAs.

i) 2 bp insertion + 44 bp deletion: c.217_219insAA + c.211_255delCCCGCTACAGCATCCGGGA GAATGGTCAGCTCCTCACCATTCTG

ii) 3 bp deletion + 14 bp deletion: c.229_231delATC + c.243_255delCAGCTCCTCACCAT

iii) 2 bp deletion: c.233_235delGG

iv) 10 bp deletion: c.231_240delCGGGAGAATGGTC

From the 4 variants detected in the KO-1 cell line, three originated a frameshift mutation leading to a premature stop codon and one caused a missense mutation (p.T73K) followed by a deletion of 14 aminoacids (p.R74_L87del) **(Figure S3)**. Since the KO-1 cell line did not generate AChR clusters after agrin incubation, it was thought that the allele containing the in frame deletion is non-functional.


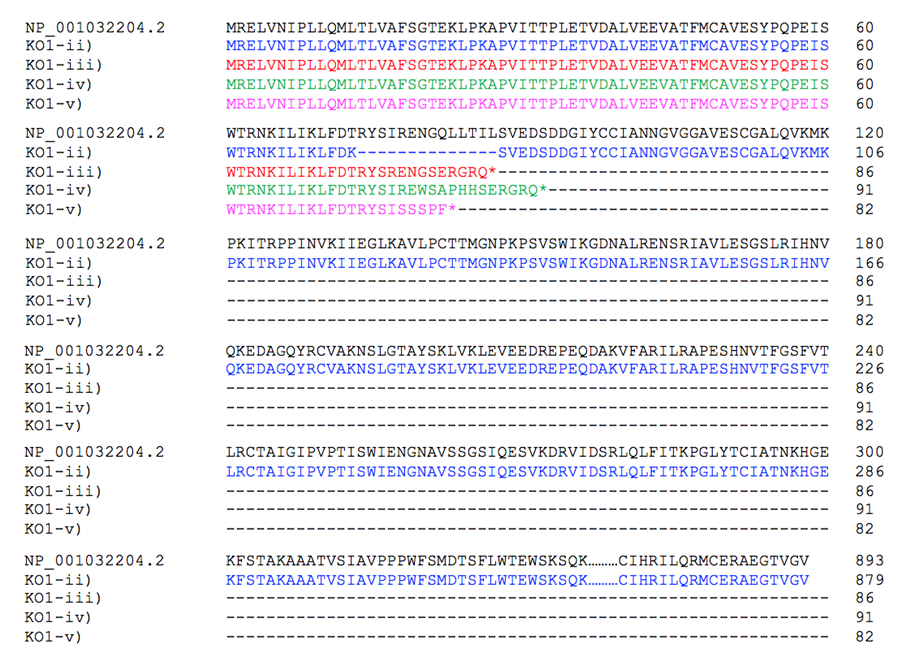


Figure S3. Alignment of amino acid sequences from mutant MuSK KO-1 alleles to WT sequence (NP_001032204.2). The mutations identified in the alleles iii), iv) and v) from KO-1 cell line shifted the coding sequences of MuSK resulting in the truncation of the protein. The mutation identified in the KO-1 ii) allele resulted in a missense mutation (p.T73K) followed by a deletion of 14 aminoacids (p.R74_L87del).

### MuSK protein expression in C2C12 Musk KO clonal cell lines

To confirm that the mutations described above caused disruption of MuSK protein, cell lysates from control C2C12 and KO-1myotubes were analysed by western blotting with antibodies against MuSK and α-tubulin. According to the manufacturer, the R&D AF-562 anti-MuSK antibody was developed using mouse myeloma cell line NS0-derived recombinant rat MuSK. The results showed non-detectable MuSK protein expression in the *Musk* KO cell lines, thus confirming the absence of MuSK **(Figure S4)**.

**Figure S4. Western blot of MuSK expression in C2C12 WT and *Musk* KO myotubes.** Lysates from mouse C2C12 WT and *MusK* KO cell lines were prepared, resolved by SDS-PAGE, and analysed by western blotting using antibodies against MuSK and α-tubulin. HEK-293T cells transiently transfected with expression vectors encoding human MuSK transcript variant 1 (Refseq NM_005592) and pcDNA3.1/Hygro(+) were used as controls.

### Quantification of AChR clusters

Images from C2C12 myotubes cultures incubated with full-length agrin and Alexa Fluor® 594**-**conjugated α-bungarotoxin were taken using an Olympus IX71 fluorescence microscope and Simple PCI software (Digital Pixel Imaging Systems, Brighton, UK). Images were acquired using a 20x magnification objective. An ImageJ macro was developed for the automated quantification of number, length, area and intensity of AChR clusters per microscope field on ImageJ version 2.0.0-rc54/1.51g **(Figure S5)**. To ensure the specificity of the procedure, the parameters of the macro (threshold, particles size, and sigma) were adjusted for each experiment using a “No agrin” control sample where AChR clusters were not present or *Musk* KO myotubes incubated with full-length agrin overnight. **(Figure S6).**


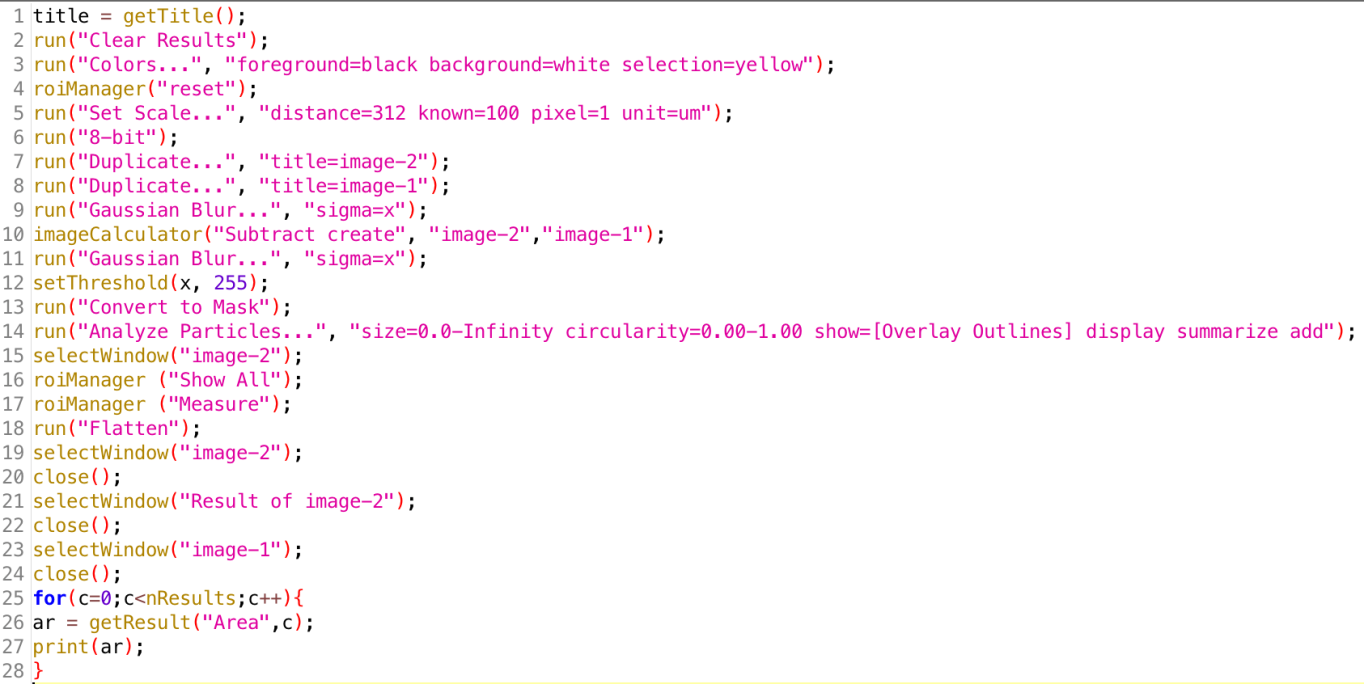
 **Figure S5**. ImageJ macro commands for the analysis of AChR clusters in C2C12 myotubes

FigureS6. Representative images of ImageJ macro analysis of AChR clusters in C2C12 myotubes (upper panel) and C2C12 *Musk*-KO cells (lower panel).

**Figure S7. Representative images of AChR clusters in C2C12 *Musk* KO cells overexpressing human MuSK WT**

**Figure S8. Representative images of AChR clusters in C2C12 *Musk* KO cells overexpressing human MuSK A617V**

Figure S9. Representative images of AChR clusters in C2C12 *Musk* KO cells overexpressing human MuSK C317R

**Protein stability and protein interaction assays for MuSK WT and A617V**

MuSK protein expression relative to baseline did not show significant differences between MuSK WT and p.A617V after incubation for 12 and 24 hours with cycloheximide at 20 µg/ml **(Figure S10)**. With regards to protein interaction with DOK7 and LRPR4, co-immunoprecipitation assays showed no major effect of p.A617V on the amount of LRP4 or DOK7 being pulled-down **(Figure S11)**.

***
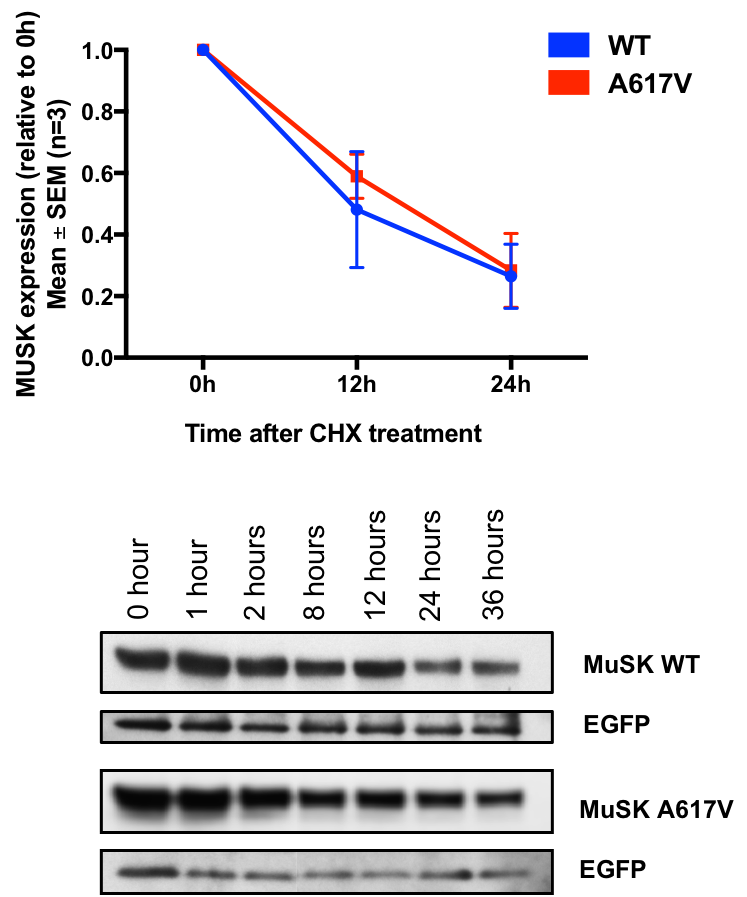
***

Figure S10. Stability of MuSK WT and p.A617V proteins

HEK-293T cells were cotransfected with WT or mutant human MuSK expression vectors, and pEGFP_N1 to control for transfection efficiency. Thirty-six hours after transfection, the cells were exposed to cycloheximide 20 μg/ml and incubated for 0, 1, 2, 4, 8, 12 or 24 hours. Subsequently, the cells were extracted and resolved by SDS-PAGE. Immunoblotting was performed with goat polyclonal anti-MuSK antibody (R&D – Cat. No. AF562), mouse monoclonal anti α-tubulin (Sigma-Aldrich, Cat. No. T75168) and HRP-conjugated secondary antibodies. The results are shown as mean ± SEM relative to WT from n=3 experiments. One-way ANOVA and Dunnett´s multiple comparisons test.


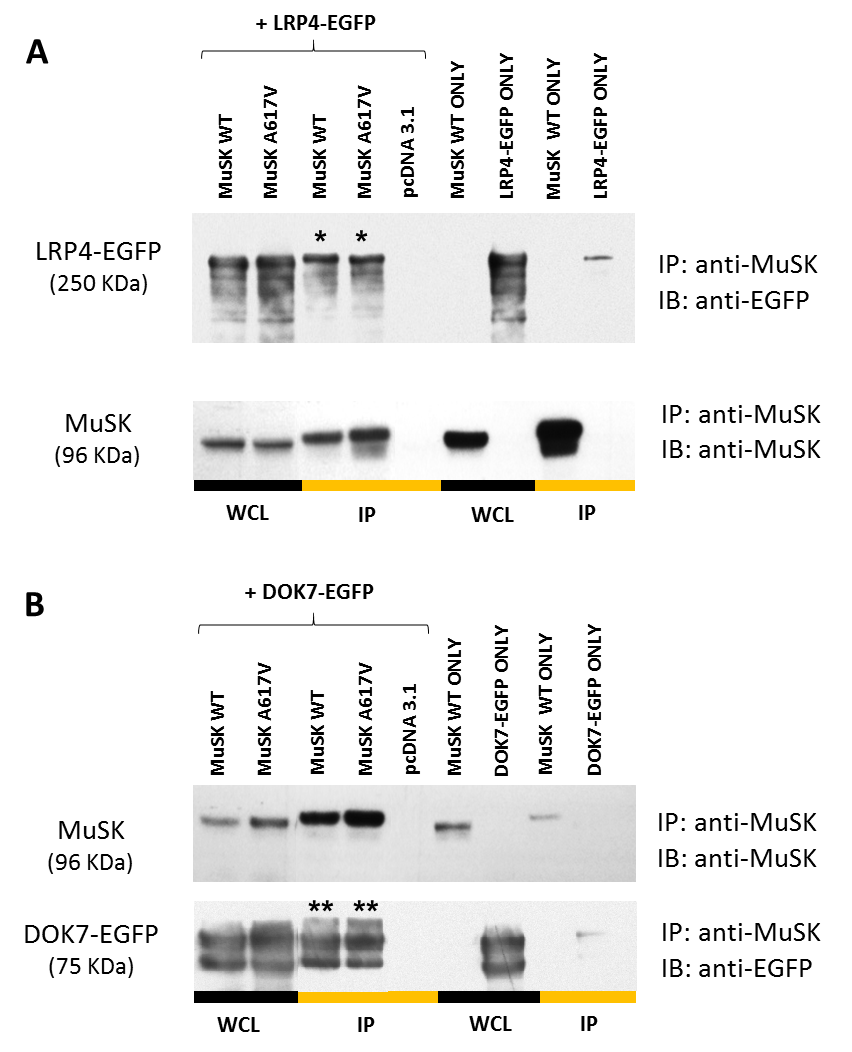


Figure S11. Coimmunoprecipitation assays of MuSK WT and variants with LRP4 and DOK7

**(A)** LRP4-EGFP was successfully co-immunoprecipitated from the surface of HEK-293T cells expressing both MuSK and LRP4-EGFP using an anti-MuSK antibody and detected with anti-EGFP antibody. MuSK p.A617V had no effect on the amount of LRP4-EGFP being pulled-down (*). WCL were used as loading controls. **(B)** DOK7-EGFP was successfully co-immunoprecipitated from the surface of HEK-293T cells expressing both MuSK and DOK7-EGFP with an anti-MuSK antibody and detected with an anti-EGFP antibody. MuSK p.A617V had no effect on the amount of DOK7-EGFP being pulled-down (**). A weak signal was observed in the LRP4-EGFP and DOK7-EGFP only lanes in probable relation with non-specific binding of EGFP to the beads. We believe this is unlikely to affect results interpretation.

**MuSK phosphorylation assays in HEK-293T cells**

No differences in tyrosine phosphorylation between MuSK WT and MuSK p.A617V were observed when MuSK was co-expressed with DOK7-EGFP or when MuSK was expressed alone in HEK-293T cells (**Figure S12).**

***
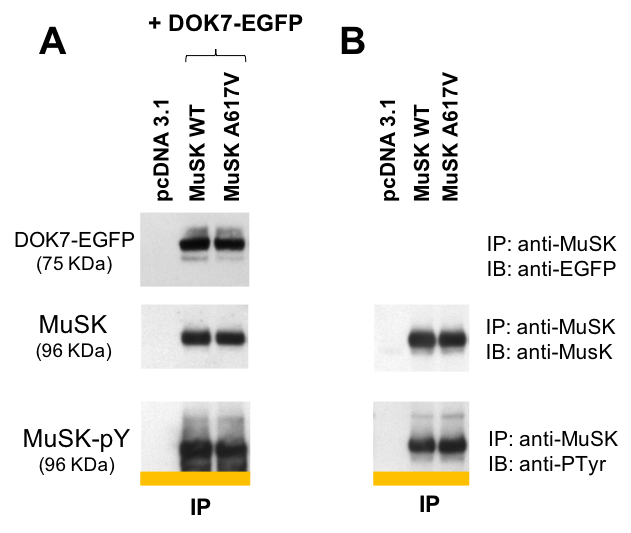
***

Figure S12. Effect of MuSK A617V on tyrosine phosphorylation in HEK-293T cells

HEK-293T cells were co-transfected with the above constructs. Subsequently, cell lysates were coimmunoprecipitated with an anti-MuSK antibody, and analysed by western blotting with antibodies to P-Tyr, MuSK and EGFP. **(A)** Representative images of western blots show lack of obvious differences in tyrosine phosphorylation between MuSK WT and p.A617V when co-expressed with DOK7-EGFP. **(B)** Representative images of western blots show lack of obvious differences in tyrosine phosphorylation between MuSK WT and p.A617V when only MuSK is transfected.

**MuSK tyrosine phosphorylation assays in C2C12 muscle cells**

Agrin-induced MuSK tyrosine phosphorylation was nullified in MuSK p.C317R myotubes in agreement with the lack of agrin-induced AChR clusters previously shown **(Figure S13)**.

**
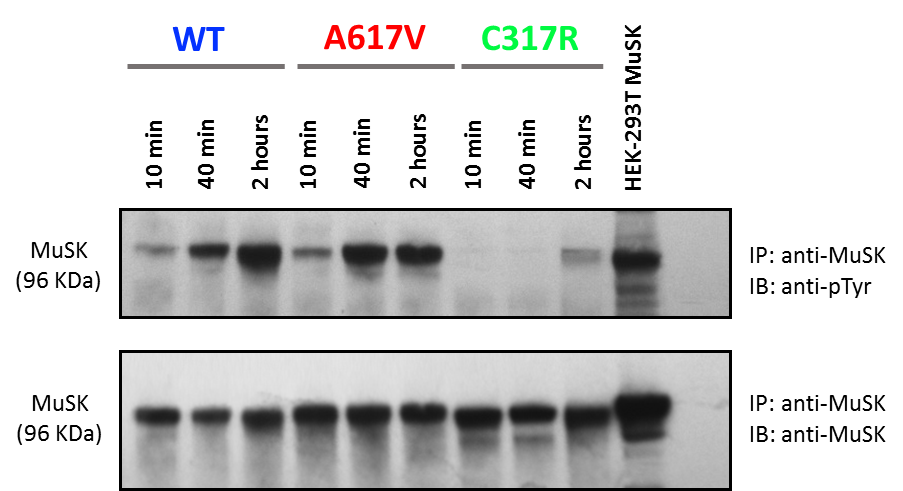
**

**Figure S13: Effect of MuSK C317R on tyrosine phosphorylation**

C2C12 *MuSK* KO myotubes infected with retrovirus expressing WT and mutant MuSK constructs were incubated with full-length agrin. Cell lysates were prepared, immunoprecipitated with anti-MuSK antibodies and analysed by western blotting with antibodies to phosphotyrosine and MuSK. The results show that MuSK phosphorylation was nullified in C317R myotubes. HEK-293T cells transfected with a WT MuSK construct were used as a positive control.

**MuSK serine phosphorylation assays in C2C12 muscle cells and HEK-293T cells**

No differences in Ser751 phosphorylation levels between WT and p.A617V myotubes were observed after incubation with full-length agrin for 8 hours **(Figure S14A)**. This specific time point was chosen based on the previous results of MuSK tyrosine phosphorylation from the agrin-induced temporal activation profile in muscle cells. In addition, there were no differences in Ser751 phosphorylation levels between MuSK WT and p.A617V in HEK-239T cells **(Figure S14B)**.


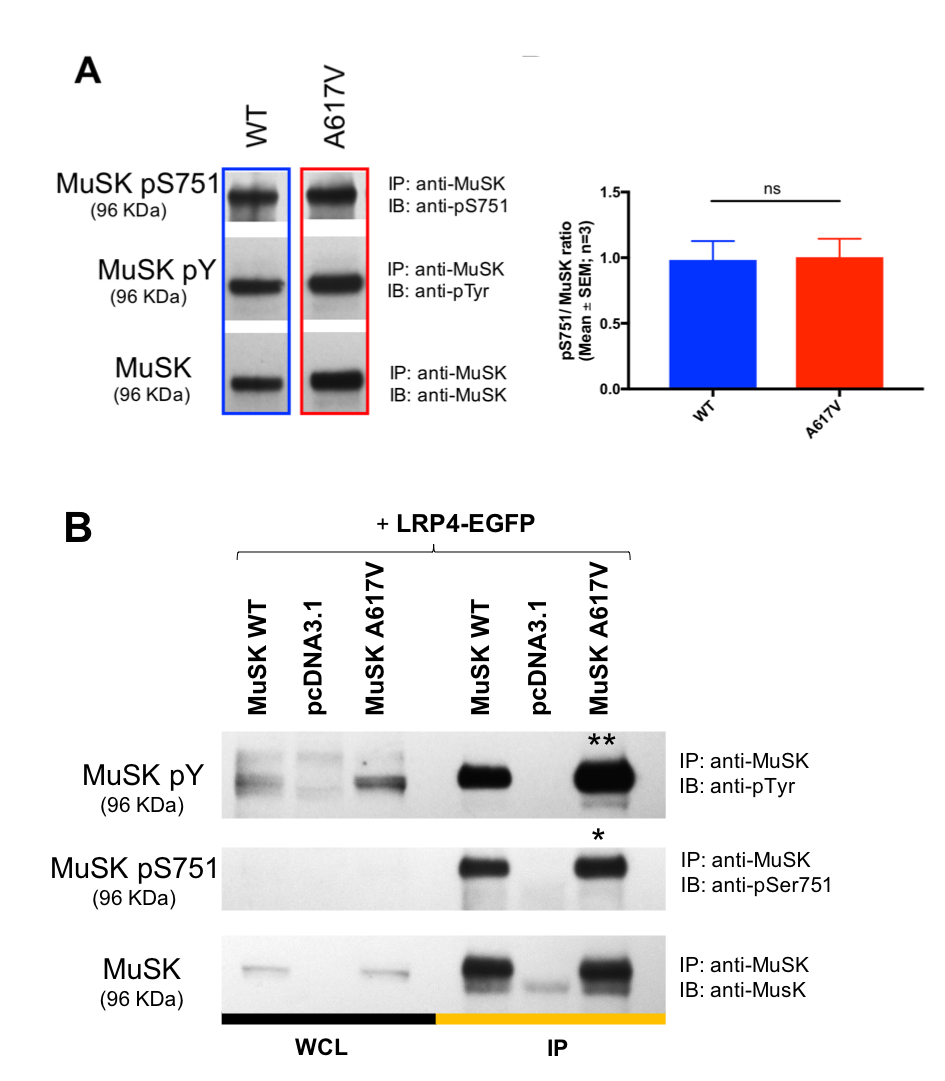


Figure S14. Effect of MuSK A617V on Ser751 phosphorylation

**(A)** C2C12 *MuSK* KO cells infected with retrovirus expressing human WT and p.A617V MuSK constructs were incubated with full-length agrin for 8 hours. No differences were observed in Ser751 phosphorylation between MuSK WT and mutant. Bar graphs represent the pS751/MuSK ratio in WT and A617V myotubes. Results are given as mean ± SEM relative to WT from n=3 experiments. Two-tailed unpaired t-test (ns = non-significant). **(B)** The results in HEK-293T cells showed lack of differences in Ser751 phosphorylation (*) between MuSK WT and p.A617V despite increased tyrosine phosphorylation (**).

**Molecular modelling of MuSK kinase domain dimerisation**

Alignment of MuSK-KD with the symmetric dimer of IGF1R-KD (Cabail et al., 2015). MuSK p.Ala617 residues are exposed to a void in the crystal and there are no obvious effects on the structure derived from the p.A617V substitution **(Figure S15)**.


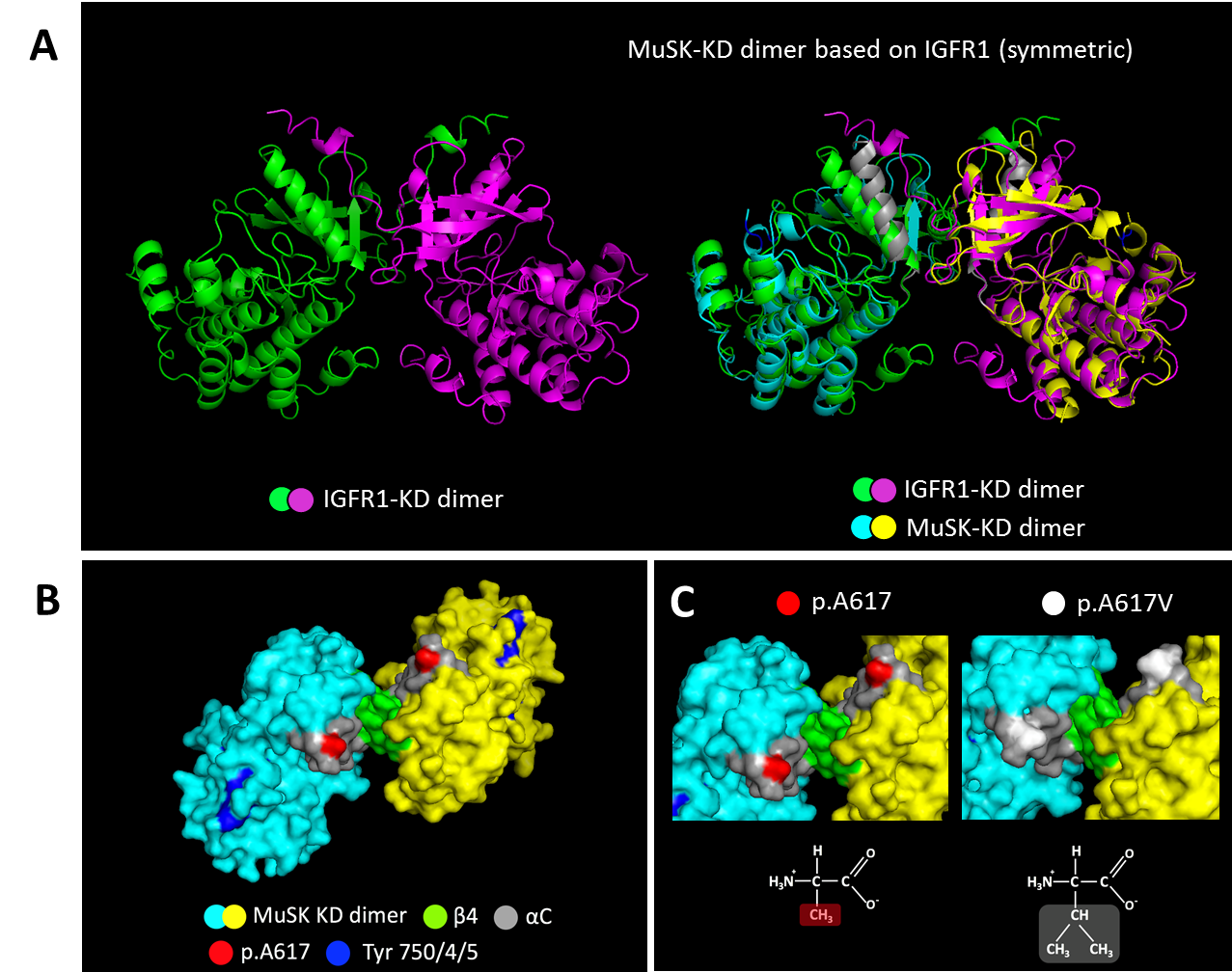


Figure S15. **Molecular modelling of MuSK KD dimerisation based on the IGF1R KD dimer structure.** **(A)** MuSK KD symmetric dimer built by homology modelling based on the crystal structure of the Insulin-like growth factor 1 receptor (IGFR1) KD dimer (MMDB ID: 128136). **(B)** Surface representation of MuSK KD dimer (cyan and yellow) based on the IGFR1 dimer. In the proposed structure, MuSK-KDs, coloured cyan and yellow, form a symmetric dimer where the αC helix (grey) from one monomer binds to the juxtamembrane region (αB) of the contralateral monomer at the N-terminus (green). In this model, the p.Ala617 residues (red) are exposed to a void in the crystal. The tyrosines within the activation loop (Tyr-750/754/755) are shown in blue. **(C)** The figure shows how the substitution of Alanine to Valine (p.Ala617Val) does not generate any obvious structural effects on the molecule.

**Response to treatment with ß2-adrenergic agonists**

Response to treatment with oral salbutamol observed in the patient **(Figure S16)**. A full video is available at the journal website.


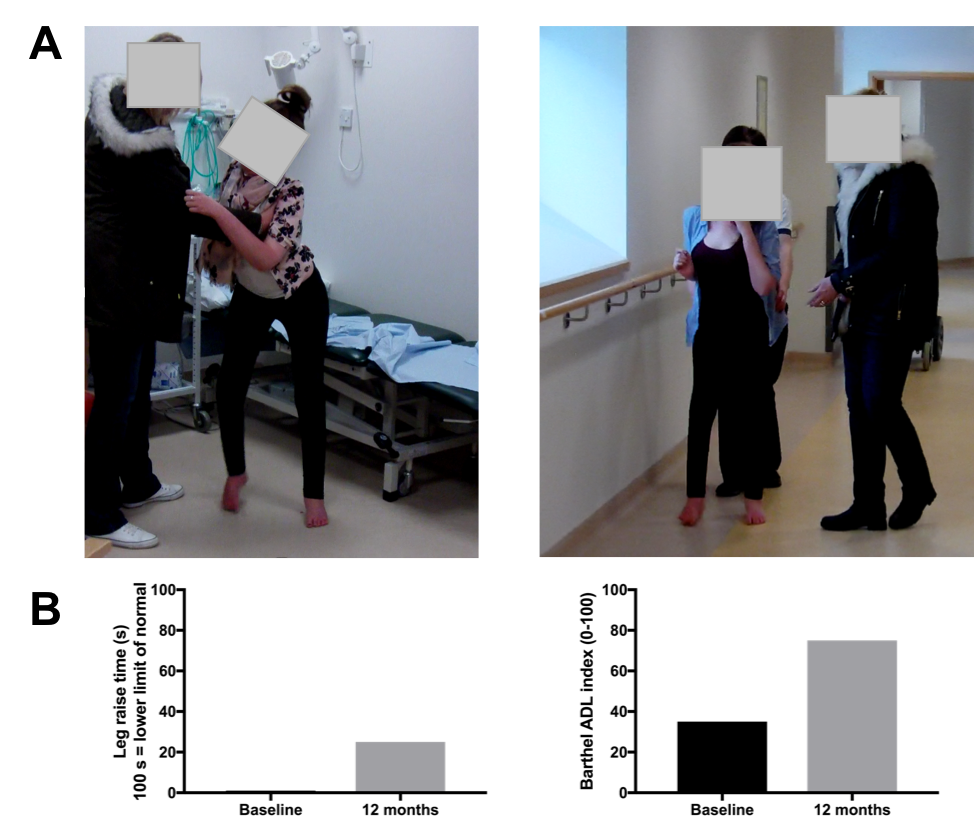


Figure S16. Clinical response to treatment with ß2-adrenergic agonists

**(A)** Representative images of the patient before (left) and after (right) treatment. **(B)** Measurement of leg rise time and Barthel Activities of Daily live index before and after treatment.

**Predicted location of Met835 in the asymmetric model of MuSK kinase domain dimer**

Alignment of MuSK-KD with the asymmetric dimer of EGFR-KD (Cabail et al., 2015). MuSK p.Met835 is located to the dimerisation interface and interacts with the p.Leu560 residue at the juxtamembrane domain from the contralateral monomer **(Figure S17)**.


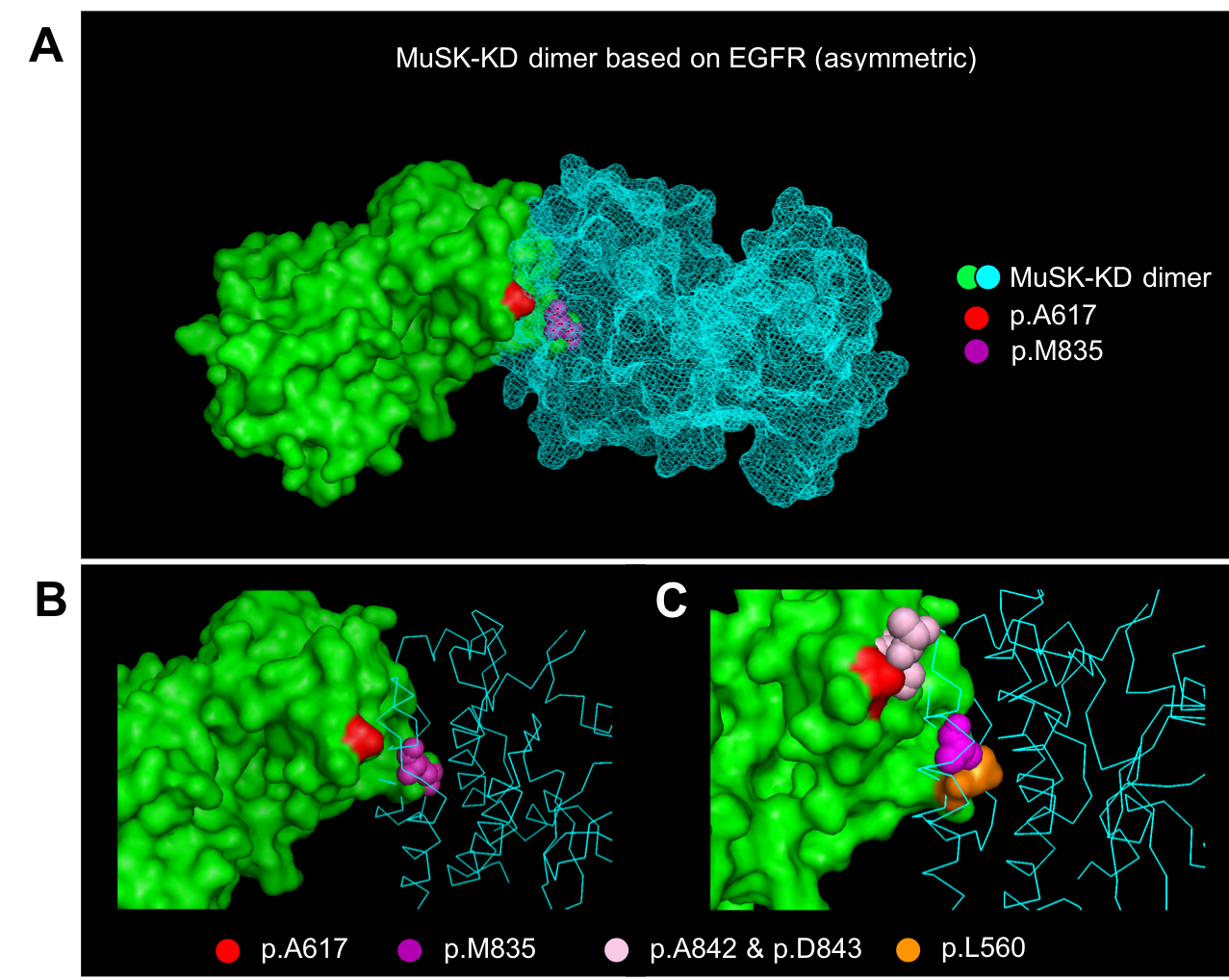


Figure S17. **Molecular modelling of MuSK KD dimerisation based on the EGFR KD dimer structure.** **(A)** MuSK KD asymmetric dimer built by homology modelling based on the crystal structure of the Epidermal Growth Factor Receptor (EGFR) KD activated dimer (MMDB ID: 40024). MuSK monomers are shown in green an cyan using surface and mesh representation, respectively. The p.Met835 residue is located within the dimerisation interface (purple spheres). **(B, C)** Detailed view of p.Ala617 and p.Met835 location and predicted interaction with residues form the contralateral monomer: p.Met835 is predicted to interact with p.Leu560 (orange) at the juxtamembrane domain and p.Ala617 is predicted to interact with p.Ala842 and p.Asp843 from the contralateral H-helix.

**Table S1: in-silico prediction of pathogenicity of *MUSK* missense variants.**

| ***MUSK* variant** | **PolyPhen-2**  **(score)** | **Mutation Taster 2**  **(score)** |
| --- | --- | --- |
| NM_005592.3:c.949T>C;(p.C317R) | Probably damaging  (1.000) | Disease causing  (180) |
| NM_005592.3:c.1850C>T;(p.A617V) | Possibly damaging  (0.880) | Disease causing  (64) |

Polyphen-2 is available online on <http://genetics.bwh.harvard.edu/pph2/>. Mutation Taster 2 is available online on <http://www.mutationtaster.org/>. dbNSFP 4.0 compiles the prediction scores from different algorithms and conservation scores and is available online on <https://sites.google.com/site/jpopgen/dbNSFP>. The results from this more comprehensive software are included supplementary data in a separate spreadsheet.
